# Translational strategy to support the first-in-human study of a TCR-like T cell bispecific with an *in vitro*-based safety approach

**DOI:** 10.3389/fimmu.2026.1736584

**Published:** 2026-04-17

**Authors:** Angelique Augustin, Blandine Avignon, Christophe Boetsch, Ekaterina Breous-Nystrom, Olaf Broders, Lauriane Cabon, Karen Dernick, Evelyne Durr, Miro Julian Eigenmann, Susanne Fischer, Nick Flinn, Regine Gerard, Anna Maria Giusti, Nikolche Gjorevski, Hans Juergen Grote, Fabian Häusermann, Nina Hobi, Elisabeth Husar, Laurent Juglair, Simon Patrick Keiser, Nino Keshelava, Stefan Kustermann, Celine Marban-Doran, Estelle Marrer-Berger, Iris Martinez Quetglas, Virginie Micallef, Ramona Matheis, Daniela Ortiz Franyuti, Liudmila Polonchuk, Giulia Raggi, Andreas Roller, Pamela Ruffiner, Salma Sadok, Tulun Saylan, Nathalie Schaub, Desirée Schubert, Satyajit Shetage, Nadine Stokar-Regenscheit, Antje-Christine Walz, Tina Weinzierl, Vincent Wolowski, Christine Zihlmann

**Affiliations:** 1 Roche Pharma Research and Early Development, Roche Innovation Center Basel, F. Hoffmann-La Roche, Basel, Switzerland; 2 Roche Pharma Research and Early Development, Roche Innovation Center Zurich, Roche Glycart AG, Schlieren, Switzerland; 3 Roche Institute of Human Biology, Roche Innovation Center Basel, F. Hoffmann-La Roche, Basel, Switzerland; 4 Roche Early Development Safety, Roche Products Ltd., Welwyn Garden City, United Kingdom; 5 Roche Pharma Research and Early Development, Roche Innovation Center Munich, Roche Diagnostics GmbH, Penzberg, Germany; 6 AlveoliX AG, Swiss Organs-on-Chip Innovation, Bern, Switzerland; 7 Roche Pharma Development, Regulatory, Roche Products Ltd., Welwyn Garden City, United Kingdom; 8 Roche Pharma Development, Data Sciences, F. Hoffmann-La Roche, Basel, Switzerland

**Keywords:** first-in-human (FIH) dose prediction, *in vitro* models, MAGE-A4/HLA-A*02:01, new approach methodologies (NAMs), nonclinical safety evaluation, TCR-like T cell bispecific (TCB), translational PKPD, organoids

## Abstract

The nonclinical development of T-cell receptor (TCR)–based therapeutics is uniquely challenged by the human-exclusive specificity of the peptide-HLA target, rendering conventional animal models unsuitable for safety assessment. To support first-in-human (FIH) studies in patients with solid tumors with the novel TCR-like T Cell Bispecific (TCB) antibody MAGE-A4-TCB, targeting the MAGE-A4 peptide presented by HLA-A*02:01 and engaging T-cells via the CD3ϵ chain, we implemented a comprehensive, prediction-independent safety strategy relying on New Approach Methodologies (NAMs). This approach utilized 21 human cellular systems, including 2D and 3D *in vitro* models of vital organs (such as liver, heart, kidney, and lung), co-cultured with allogeneic peripheral blood mononuclear cells to identify potential off-target toxicity and a whole blood assay for cytokine release risk assessment. The concentration-effect relationship and Minimal Anticipated Biological Effect Level (MABEL) were established using the A375 cancer cell line, chosen for its comparable sensitivity to ovarian carcinoma organoids, which were the most sensitive out of 22 tested tumor organoids. Nonclinical testing demonstrated an absence of off-target reactivity in vital organs, though the whole blood assay predicted cytokine release syndrome as the primary risk. The calculated FIH starting dose was safe in the subsequent Phase I study (NCT05129280), and the most commonly reported adverse events were treatment-related skin and subcutaneous tissue disorders (73% of patients). These skin-related adverse events, which appeared in contradiction to the initial *in vitro* results in skin co-culture models, were hypothesized to be driven by the target-independent activation capability of the UCHT1-based CD3 binder, potentially enhanced in patients with a systemic pro-inflammatory profile. This study provides a translational framework for TCR-based immunotherapies, demonstrating the successful application of NAMs to define a safe starting dose while highlighting the critical need for refining these methodologies to accurately predict complex, systemic immune-related adverse events, particularly those involving barrier organs.

## 1 Introduction

Despite recent advancements in survival with the introduction of immune checkpoint inhibitors (CPIs) in many solid tumors, the majority of patients progress rapidly and only a fraction of patients derive long-term benefit (1, 2). Soluble bispecific T cell redirectors, consisting of antigen recognition and T cell–engaging domains, have been proposed to overcome resistance to CPI by redirecting the immune system to eliminate otherwise non-immunogenic tumor cells. Development for solid tumors has been challenging, largely because of the paucity of truly tumor-restricted surface antigens and additional obstacles specific to solid malignancies (e.g., T-cell infiltration, immunosuppressive tumor micro-environment) (3). T-cell receptor (TCR)–based therapeutics expand the target space by recognizing intracellular peptides presented on MHC class I (pHLA/pMHC) and can be implemented either as engineered soluble TCRs (ImmTACs) or as TCR-like (TCR-mimic) antibodies (4–6). The approval of tebentafusp (targeting a peptide derived from the intracellular oncoprotein gp100) for HLA-A*02:01-positive uveal melanoma proves that pHLA-redirecting approaches can mediate meaningful clinical benefit in solid tumors (7, 8). Nonclinical development of TCR-based therapeutics is especially demanding because these agents carry a higher risk of off-target/cross-reactivity liabilities compared with conventional T-cell bispecifics that bind membrane proteins (6, 9) and are human-specific (pHLA epitopes and HLA restriction), limiting the usefulness of standard animal models (10, 11). Clinical fatalities and severe toxicities observed with affinity-enhanced TCRs and adoptive T-cell therapies (for example, the MAGE-A3/Titin cross-reactivity and other neuro-/cardiotoxic events) underscore the need for rigorous, tailored preclinical safety packages that detect off-target reactivity prior to first-in-human studies (12–14). The strict human specificity of the MHC-I molecules, together with the limited overlap in protein processing and resulting peptides, compromise the use of animal models for safety testing and assessments. Regulatory guidance (ICH S6/R1 and ICH S9 context (15, 16)) indicates that when no pharmacologically relevant species exist, robust *in vitro* human-based assessments should be considered to support FIH dosing and safety decisions (11). Here we describe a translational strategy used to predict the safety, starting dose and efficacious dose range for a novel TCR-like TCB which targets T−cells (via CD3ϵ) and an intracellular peptide derived from the melanoma-associated antigen A4 (MAGE-A4) presented by HLA-A*02:01 (via the MAGE-A4/HLA-A*02:01 complex). Simultaneous binding of MAGE-A4-TCB to both targets is expected to lead to T-cell activation and subsequent killing of cancer cells expressing MAGE-A4 and presenting MAGE-A4-derived peptide on the cell surface. MAGE-A4 is a cancer/testis antigen with restricted expression in normal immune-privileged tissues and broad expression across several solid tumors, making it an attractive pHLA target (17–20).

The approach presented here focuses on the *in vitro*/ex vivo testing of the clinical candidate MAGE-A4-TCB using human model systems of healthy and diseased tissues/cells to support First-in-Human (FIH) studies. Thereafter, the manuscript presents the clinical experience with the same candidate.

## 2 Materials and methods

### 2.1 Therapeutic molecule and controls

**Figure 1:**
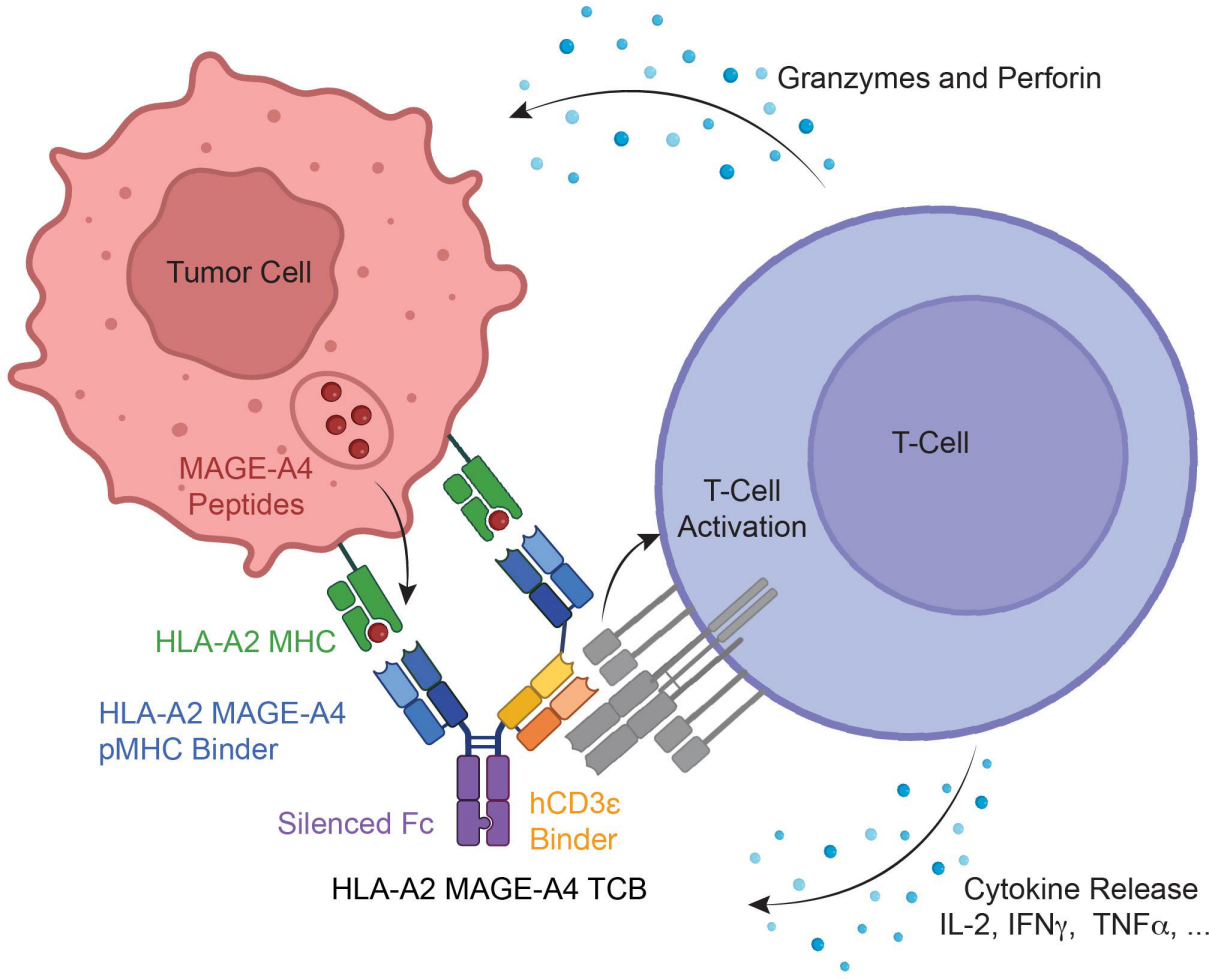
Schematic illustration of the structure and mechanism of action of the MAGE-A4-TCB antibody. MAGE-A4-TCB is a 2 + 1 humanized IgG1 antibody targeting the human MAGE-A4{230-239} peptide (GVYDGREHTV) in complex with HLA-A*02:01. The antibody engages T-cells via a single V9 Fab fragment (UCHT1-derived) specific for the human CD3ϵ chain. The Fc region contains P329G LALA mutations to prevent target-independent activation through FcgR binding. Simultaneous binding to tumor-expressed pMHC and T-cell CD3 mediates T-cell activation, degranulation, and subsequent tumor cell lysis. hCD3e, human CD3 ϵ chain; MAGE-A4, melanoma-associated antigen 4; pMHC, peptide-major histocompatibility complex; TCB, T-cell bispecific antibody; VH, variable domain of heavy chain; VL, variable domain of light chain.

MAGE-A4-TCB is a bispecific humanized monoclonal antibody based on a human immunoglobulin G1 (IgG1). Two human Fab domains bind to human MAGE-A4230-239 (GVYDGREHTV) peptide-major histocompatibility complex (pMHC) of the human leukocyte antigen (HLA)-A*02 allotype on MAGE-A4 positive tumor cells (21). One humanized Fab domain binds to the human CD3ϵ chain on T−cells (**Figure 1**). Correct chain association is enabled by the CrossMabVH-VL technology combined with charge interactions for the light chains and knob-into-holes technology for the heavy chains (21, 22). Fc effector functions are abolished by introducing P329G LALA mutations into the Fc portion (23). By virtue of its selectivity for the human HLA-A*02 allotype, the MAGE-A4pMHC binder is human specific as it cannot recognize MAGE-A4230–239 peptide bound to MHC molecules from other species. The CD3 binding arm is based on the humanized V9 antibody, which is derived from the CD3ϵ-specific murine antibody UCHT1 (24); like UCHT1, it binds to a conformational epitope of the CD3ϵ chain and does not cross-react with the cynomolgus monkey or mouse species (25).

The following controls were utilized:

- DP47-TCB (Negative Control): A non-targeted 2 + 1 TCB possessing the same V9 CD3 binder but with two non-binding germline DP47 Fabs to monitor target-independent T-cell activation.
- ESK-1-TCB (Positive Control): A non-selective TCRL-TCB recognizing multiple peptide-HLA-A*02 complexes, used to validate effector and target system responsiveness.
- Reference Toxicity Controls: Chlorpromazine (Cat: C2481, Sigma-Aldrich), Doxorubicin (Cat: 15007, Cayman Chemical), and Dofetilide (Cat: 15063, Cayman Chemical).

### 2.2 Retrogenix screen

Specific off-target binding of the bispecific human IgG1 antibody MAGE-A4-TCB (test antibody) was assessed using Retrogenix Cell Microarray Technology. The library consisted of 6,101 individual expression vectors encoding full-length human plasma membrane proteins, secreted proteins, and cell surface-tethered human secreted proteins, plus 396 human heterodimers. These were individually arrayed in duplicate across cell microarray slides and reverse-transfected into human HEK293 cells. Following cell fixation, MAGE-A4-TCB was added at a screening concentration of 20μg/mL. Binding was detected using an AlexaFluor647-conjugated anti-PGLALA secondary antibody (5μg/mL), and fluorescent images were analyzed via ImageQuant software. A protein interaction was defined as a duplicate spot showing a raised signal compared to background levels upon visual inspection. In a confirmatory screen (Fixed and Live Cells) vectors encoding all 27 identified library interactions were re-arrayed along with a requested receptor (CD3E) and a control receptor (EGFR). Confirmation was performed on both fixed cells (n=2) and live cells (n=1). Slides were treated with 20ug/mL of MAGE-A4-TCB, 20μg/mL of the positive control antibody DP47 TCB, or secondary antibody alone as a negative control. Interactions were classified by intensity as strong, medium, weak, or very weak; “significant” interactions were defined as those with weak intensity or greater.

### 2.3 Tissue cross-reactivity study (normal human tissues)

A GLP tissue cross-reactivity study in normal human tissues was performed, in order to determine the potential-cross reactivity of MAGE-A4-TCB. MAGE-A4-TCB, was applied to cryosections of normal human tissues (n ≥ 3 donors/tissue) at two concentrations (15 and 5 μg/mL). The human test tissues included in the study were positive for НLA-A2 (at least 2 of the 3 donors per tissue, as available). In addition, the test article was substituted with a monoclonal human IgG1 antibody that binds only to CD3E, designated DP47-TCB.

### 2.4 Effector cell selection and ranking

Peripheral blood mononuclear cells (PBMCs) from healthy HLA-A*02:01-positive donors were used as effector cells. Donor functional activity was pre-characterized by stimulating cells with a CD20-TCB for 18 hours then incubated with a protein transport inhibitor for 6 hours. Intracellular cytokine expression and immune cell activation markers (IFNγ, TNFa, CD107a, Granzyme B, CD69, CD25, HLA-DR, IL17, IL6, IL8, IL1b) were measured by Flow Cytometry using a T cell specific panel and a leukocyte panel and all conditions ran in triplicates.

This data was used to create a composite score of 33 parameters selected based on their correlation with an effect on the target cell (B cells) killing, including 25 population markers out of 75 from the T cell panel and 8 population markers out of 49 in the leukocyte panel.

T cell panel parameters used for the scoring: %_CD8+_of_live_cells, ratio_CD8+_CD4+, %_CD45RA-_CCR7-_TEM_of_CD8+_cells, % CD107a+ GranzymeB+_of_CD8+_cells, %_GranzymeB+_of_CD8+_cells, %_CD107a+_of_CD8+_cells, % IFNγ+_TNFa+_of_CD8+_cells, %_IFNγ+_of_CD8+_cells, %_TNFa+_of_CD8+_cells, %_HLADR+_of_CD8+_cells, % CD25+_CD69+_of_CD8+_cells, %_CD25+_of_CD8+_cells, %_CD69+_of_CD8+_cells, %_CD45RA-_CCR7-_TEM_of_CD4+_cells, %_IL17a+_Th17_of_CD4+_cells, % CD107a+_GranzymeB+_of_CD4+_cells, %_CD107a+_of_CD4+_cells, %_GranzymeB+_of_CD4+_cells, %_CD69+_of_CD4+_cells, %_HLADR+_of_CD4+_cells, %_CD69+_HLADR+_of_CD4+_cells, %_IFNγ+_of_CD4+_cells, %_TNFa+_of_CD4+_cells, %_IFNγ+_TNFa+_of_CD4+_cells, %_IFNγ+_of_DNTc_cells.

Leukocyte panel parameters used for the scoring: %_IL8+_of_CD14+_monocytes, %_IL1beta+_of_CD14+_monocytes, %_IL6+_of_CD14+_monocytes, %_IFNγ+_of_CD14+_monocytes, %_TNFa+_of_CD14+_monocytes, %_IFNγ+_TNFa+_of_CD19-_CD20-_CD3-_CD33-_”NK”_cells, %_IFNγ+_of_CD19-_CD20-_CD3-_CD33-_”NK”_cells, %_TNFa+_of_CD19-_CD20-_CD3-_CD33-_”NK”_cells.

For the safety screening panel, three donors were selected: Athens (Mid-responder), Vienna (High-responder), and Dublin-4 (High-responder).

### 2.5 Description of experimental systems and culture conditions

Off-target reactivity was evaluated across 21 human cellular systems representing human vital organs. All target cells were verified for HLA-A*02:01 haplotype prior to use.

#### 2.5.1 2D primary cell models

Primary cells were cultured in specialized growth media and seeded in flat-bottom 96-well plates 24 hours prior to co-culture to allow for adherence.

- Integumentary and Cardiovascular Systems: Normal Human Epidermal Keratinocytes (NHEK), Melanocytes (NHEM), Aortic Endothelial Cells (HAEC), and Microvascular Endothelial Cells (HMVEC-C) were seeded at 20,000 cells per well.
- Respiratory and Renal Systems: Small Airway Epithelial Cells (SAEC) (Cat: CC-2547, Lonza), Bronchial Epithelial Cells (NHBE) (Cat: CC-2540, Lonza), and Renal Proximal Tubule Epithelial Cells (RPTEC) (Cat: CC-2553, Lonza) were seeded at 20,000 to 30,000 cells per well.
- Neurological System: Human LUHMES neurons were seeded at 50,000 cells per well on plates coated with poly-L-ornithine (Cat: P3655, Sigma-Aldrich) and fibronectin (Cat: F1141, Sigma-Aldrich). Human iPSC-derived astrocytes (Cat: R1092, Cellular Dynamics) were seeded at 20,000 cells per well on Matrigel-coated plates (Cat: 354230, Corning).

#### 2.5.2 3D microphysiological and organoid systems

Liver Spheroids: 3D microtissues consisting of primary human hepatocytes and non-parenchymal cells at a 2,000 to 1,000 ratio were cultured in InSphero GravityTRAP plates (Cat: CP-09-001, InSphero).

Intestinal Organoids: Patient-derived duodenum and colon organoids were embedded in 50 percent Matrigel (Cat: 356231, Corning). Assays were conducted on expansion-state organoids and differentiated organoids using specialized media (Cat: 06010 and 100-0212, STEMCELL Technologies).

Pancreatic Islets: 3D InSight Islet Microtissues (Cat: MT-04-002-01-60, InSphero) composed of primary alpha, beta, and delta cells were used.

^AX^Lung-on-Chip: alveolus on chip model (AlveoliX) utilized biopsy-derived alveolar epithelial cells and human peripheral blood mononuclear cells cultured on a porous flexible membrane to simulate the thin alveolar microenvironment.

### 2.6 Co-culture standardization and inflammatory simulation

A standardized Effector to Target (E:T) ratio of 8:1 was utilized for 2D systems, comprising 160,000 PBMCs added to 20,000 target cells. On Day 0, PBMC were thawed in RPMI-1640 + 10% FBS and centrifuged at 300g for 10 minutes. PBMC were washed in a further 10ml RPMI-1640 + 10% FBS, passed through a 70uM cell strainer, and counted using a Muse Cell Analyzer (Luminex). PBMC were re-suspended in RPMI-1640 + 10% FBS and co-cultured with the target cells at a final seeding density of 160,000 PBMC/well. For 3D models, the ratio was adjusted to approximately 5:1 based on tissue-specific cell counts. Co-cultures were maintained in a 1:1 ratio of target-specific medium and PBMC medium (RPMI-1640 (Cat: 61870, Gibco) supplemented with 10% FBS (Cat: 10500, Gibco)). To simulate systemic inflammation and maximize target-presentation sensitivity, parallel cultures were pre-treated with 100 pg per mL IFN-gamma (Cat: 285-IF, R&D Systems) for 24 hours prior to drug addition.

Analytical Readouts and Flow Cytometry Analysis Assay readouts were collected at 24, 48, and 72 hours post-treatment.

#### 2.6.1 Cytotoxicity and cytokine release

Target cell lysis was quantified via LDH release using the LDH-Glo Cytotoxicity Assay (Cat: J2380, Promega) or Caspase 3/7 cleavage using the CellEvent Caspase-3/7 Green Detection Reagent (Cat: C10423, ThermoFisher). A positive response was defined as a Stimulation Index (SI) greater than 1.8 with p less than 0.05 compared to untreated controls. Supernatants were analyzed for cytokine release using a Luminex 8-plex panel (Cat: M50000007A, Bio-Rad) to detect IFN-gamma, IL-2, IL-4, IL-6, IL-8, IL-10, TNF-alpha, and GM-CSF.

#### 2.6.2 T-cell proliferation and surface staining

Cellular proliferation was measured via DNA synthesis using EdU (5-ethynyl-2’-deoxyuridine) incorporation (Cat: C10418, ThermoFisher). For 3D systems, PBMCs were pre-labeled with the CellTrace Far Red Proliferation Kit (Cat: C34564, ThermoFisher). PBMCs were harvested and prepared for multi-color flow cytometric analysis. Cells were blocked with True Stain Monocyte Blocker (Cat: 426103, BioLegend) and Human TruStain FcX (Cat: 422302, BioLegend) followed by staining with the following antibody panel:

- Lineage Markers: CD3-BV421 (Cat: 317344, BioLegend), CD4-BUV737 (Cat: 612748, BD Biosciences), CD8a-BV510 (Cat: 344732, BioLegend), CD19-BV785 (Cat: 563325, BD Biosciences), CD14-BV711 (Cat: 301838, BioLegend), and CD16/56-BUV805 (Cat: 742075, BD Biosciences).
- Activation Markers: CD69-PECy7 (Cat: 557745, BD Biosciences), CD25-BV605 (Cat: 302632, BioLegend), and CD137-PE (Cat: 309804, BioLegend).
- Viability and Discrimination: DAPI (Cat: 62248, ThermoFisher) for dead cell exclusion and EpCAM-PerCPCy5.5 (Cat: 324214, BioLegend) for target cell identification.

Samples were acquired on an LSR Fortessa X-20 (BD Biosciences). Absolute cell counts were determined using Count Bright Beads (Cat: C36950, ThermoFisher). A positive proliferative response was defined as an SI greater than 5 with p value less than 0.05.

### 2.7 Statistical analysis

All experiments were conducted in technical triplicates using at least three independent PBMC donors on two independent target cell donors. Statistical significance was evaluated using one-way ANOVA followed by Tukey’s multiple comparison test. Data normalization was performed relative to the baseline of untreated co-cultures for each specific donor and tissue system.

### 2.8 Assessment of organ-specific physiological parameters

#### 2.8.1 Liver-specific injury markers

For the 3D liver microtissue models, hepatotoxicity and cellular damage were assessed through the analysis of culture supernatants. Aspartate Aminotransferase (AST) activity was quantified using an ADVIA 1650 autoanalyzer (Bayer) according to the EC 1.1.1.27 methodology. To provide high-sensitivity detection of liver injury, microRNA-122 (miR-122) levels were determined via quantitative real-time PCR (qRT-PCR) using a QuantStudio 7 Flex Real-Time PCR System (ThermoFisher). RNA was analyzed using the TaqMan probe hsa-miR122 (Cat: 002245, ThermoFisher), with normalization performed against the ath-miR159a probe (Cat: 000338, ThermoFisher).

To distinguish between apoptotic and necrotic cell death, total and cleaved Keratin-18 levels were measured using M65 and M30 ELISA kits (Cat: 10020 and 10011, Peviva). Furthermore, acute hepatocellular injury was monitored through the measurement of alpha-Glutathione S-Transferase (alpha-GST) (Cat: NE0300, Oxford Biomedical Research) in the spheroid supernatants.

#### 2.8.2 Cardiomyocyte electrophysiology and viability

Functional cardiotoxicity and electrophysiological changes in human iPSC-derived cardiomyocytes were evaluated using the CardioExcyte 96 platform (Nanion Technologies). The cardiomyocyte beat rate was extracted from Extracellular Field Potential (EFP) recordings, providing a non-invasive measure of rhythmic activity. Simultaneously, the base impedance of the cardiomyocyte monolayer was continuously monitored as a real-time indicator of cell viability and monolayer integrity. Data acquisition was managed using the CardioExcyte Control software, and comprehensive waveform analysis was performed using the DataControl 96 software package (Nanion Technologies).

#### 2.8.3 Alveolar barrier integrity and effector interaction in ^AX^Lung-on-Chip

In the ^AX^Lung-on-Chip microfluidic models, the functional integrity of the alveolar-capillary barrier was assessed by measuring the Transepithelial Electrical Resistance (TEER) across the epithelial-endothelial interface. To study the spatial recruitment of immune cells within the microfluidic environment, PBMCs were pre-labeled with the CellTrace Far Red Proliferation Kit (Cat: C34564, Life Technologies). The attachment and distribution of effector cells on the alveolar epithelium were quantified through high-resolution fluorescence imaging of the microfluidic chips.

### 2.9 Morphological assessment and fluorescence microscopy

Morphological changes and specific biomarkers of cell death were evaluated across cellular systems using high-content and fluorescence microscopy. To monitor the localized activation of the apoptotic cascade in co-cultures, cells were incubated with the CellEvent Caspase-3/7 Green Detection Reagent (Cat: C10423, ThermoFisher). Images were acquired for qualitative assessment and quantitative analysis of target cell health and T-cell mediated effects. Automated image processing for morphological features and marker intensity was conducted to support the functional readouts of the safety screen.

### 2.10 Mass spectrometry-based immunopeptidomics and target quantification

To quantify the density of MAGE-A4 230–239 peptide HLA-A*02:01 complexes, a targeted liquid chromatography-tandem mass spectrometry (LC-MS/MS) method was employed. Cell pellets from tumor lines or patient-derived organoids were lysed in a buffer containing 1 percent NP40 (Cat: 28324, ThermoFisher) and protease inhibitors (Cat: 78440, ThermoFisher).

HLA-A*02:01 molecules were enriched via automated immunoprecipitation using the Agilent AssayMAP Bravo platform. Lysates were incubated with a biotinylated mouse anti-human HLA-A2 antibody, clone BB7.2 (Cat: 307611, BioLegend), immobilized on Streptavidin cartridges (Cat: G5496-60010, Agilent). Peptides were eluted using 0.1 percent trifluoroacetic acid (Cat: 28904, ThermoFisher).

The isolated HLA-peptides were separated by reverse-phase nano-chromatography on an EASY-nLC 1200 system (Cat: LC140, ThermoFisher) using a 75 micrometer by 25 centimeter C18 column. Analysis was performed on a Q-Exactive HF-X mass spectrometer (Cat: 0726042, ThermoFisher) operated in parallel reaction monitoring (PRM) mode. Absolute quantification was achieved using a heavy-labeled internal standard peptide (GVYDGREHT[V-V-Heavy]) (Cat: Custom, JPT Peptide Technologies). The assay established a lower limit of quantitation (LLOQ) of 0.27 femtomoles and a lower limit of detection (LLOD) of 0.09 femtomoles.

In addition, MAGEA4 mRNA expression and protein expression were estimated by RTqPCR and western blotting respectively. In short for mRNA expression, total RNA was isolated from fresh-frozen PDOs or cell lines using RNeasy Mini (Qiagen) or MagNA Pure 96 (Roche) kits, with quality assessed via Bioanalyzer (Tapestation) and Nanodrop. RNA (400–800 ng; 100 ng for *EN002*) was reverse-transcribed using SuperScript IV Vilo Master Mix with ezDNase (Invitrogen). For cell line analysis, cDNA was pre-amplified (Roche) and analyzed on a Biomark HD (Fluidigm). For organoid lines, qPCR was performed on a QuantStudio 6 (Thermo Fisher) using TaqMan Fast Advanced Master Mix. *MAGE-A4* expression (Hs00751150_s1) was normalized to a panel of housekeeping genes (*CASC3*, *MRPL19*, and *RHOA* or *ZNF207*) selected using a statistical modeling approach (Szabo et al., 2004). Samples were run in technical triplicates; those with standard deviations greater than 0.5 were excluded. Relative expression was calculated using the 2^-delta delta Ct method, using A375 cells as the positive control calibrator. For Western Blotting, flow-through fractions from HLA-A*02:01 immunoprecipitation were resolved by SDS-PAGE and transferred to PVDF membranes. After blocking in 5% skim milk (0.1% Tween-20 in TBS), membranes were incubated with rabbit monoclonal anti-MAGE-A4 (Cell Signaling, Cat: 82491) and anti-GAPDH-HRP (Santa-Cruz, Cat: sc-25778). Protein bands were detected using SuperSignal West Femto Maximum Sensitivity Substrate (Thermo Scientific) and visualized on a LAS4000 Imager (Fujifilm).

### 2.11 *In vitro* cytokine release assessment in human whole blood

The risk of systemic cytokine release syndrome (CRS) was assessed using an undiluted whole blood assay. Fresh whole blood was collected in lithium heparin tubes (Cat: 367884, BD Biosciences) from 30 healthy donors, including 14 HLA-A02:01 positive and 16 HLA-A02:01 negative individuals. Samples were processed and stimulated within 3 hours of blood withdrawal.

Whole blood (195 microliters per well) was incubated with MAGE-A4-TCB or controls at concentrations ranging from 0.05 to 50 nanomolar in 96-well polypropylene plates (Cat: 3365, Corning). Lemtrada (alemtuzumab) (Cat: 1045230, Genzyme) served as a positive control for cytokine release, while Erbitux (cetuximab) (Cat: 1052603, Merck) was used as a low-risk clinical comparator.

Following a 24-hour incubation at 37 degrees Celsius in a 5 percent carbon dioxide atmosphere, plasma was harvested by centrifugation. Cytokine concentrations for IFN-gamma, IL-2, IL-6, IL-8, and TNF-alpha were quantified using the Aushon Ciraplex imaging system (Cat: 04-0010, Aushon Biosystems) with multiplexed Cirascan kits. A quantile-based statistical approach (95 percent) was used to define positive response thresholds relative to the low-risk comparator for each donor.

### 2.12 Study of MAGE-A4 (57Do3)-muCD3 hemisurrogate in HLA-A*02:01 human transgenic mice

MAGE-A4 (57Do3)-muCD3 is a hemisurrogate TCB, containing the clinical binder to the HLA-A2 MAGE-A4 pMHC complex and a murine anti-CD3 binder.

Transgenic AAD (B6.Cg-Immp2lTg (HLA-A/H2-D) 2Enge/J) mice are homozygous mice generated at The Jackson Laboratory (JAX), USA. Five female AAD transgenic mice and 5 female C57BL/6J mice aged 8–9 weeks at start of the experiment were injected SC on study day 0 with 1E + 06 of IM-9 human multiple myeloma cell line expressing MAGE-A4 HLA-A2 pMHC complex mixed with 50% Matrigel. Three days later they were intravenously administered 1 mg/kg of the hemisurrogate antibody. Animals were controlled daily for clinical symptoms and detection of adverse events to ensure compliance with animal welfare. Three days upon test antibody injection all mice were terminated for monitoring of exposure and histopathological evaluation of selected organs: Adrenals, brain, bone marrow (femur, sternum), cecum, colon, duodenum, gall bladder, heart, ileum, jejunum, kidneys, liver, lung, lymph node (mesenteric), skeletal muscle, skin/subcutis, spleen, stomach, pancreas, thyroids, urinary bladder.

### 2.13 Pharmacokinetic modeling and MABEL-based dose prediction

An integral first part of the FIH definition is the estimation of PK parameters, which are subsequently scaled to human values. The non-specific PK evaluation was performed based on sampled PK in human FcRn tg32 mice after a 5 mg/kg intravenous dose. As this mouse model does not reflect any target mediated processes, a standard 2-compartmental model with linear elimination was used in order to estimate resulting PK parameters. The parameter estimation was performed in Monolix 2018R1 and a proportional error model was assumed. The resulting model fit was evaluated using prognostic plots, precision of parameter estimates and objective functions.

The estimated PK parameters were then scaled to human values using a common bodyweight-based allometric scaling approach (26), using 0.02 and 70 kg bodyweight for mice and human, respectively. The clearances were scaled with an exponent of 0.98 using the following equation:


$$Clearance_{human}=Clearance_{mouse}*{\left(\frac{BW_{human}}{BW_{mouse}}\right)}^{0.98}$$

Instead of scaling the volumes, typical values for antibody-based drugs of 3.1 and 4.1 L were assumed for central volume (Vc) and peripheral volume respectively (27). Human PK simulations using the resulting values were performed in Berkeley Madonna v8.3.18. A time-cumulative PKPD analysis was performed as previously described by Van De Vyver et al. (28). This approach calculates in a first step the cumulative response over time by assessing the AUCE of the *in vitro* effect timecourse data for each tested concentration. In a second step, a sigmoidal Emax model is then fitted to the newly established AUCE/concentration curves:


$$E=\;E_{0}+\;\frac{Emax\;*\;C_{TCB}^{y}}{EC50^{Y}+C_{TCB}^{y}\;\;}\;$$

Where E is the effect (in this case AUCE), Emax the maximal observed AUCE, EC50 the concentration resulting in half of the maximal AUCE, C_TCB_ is the TCB concentration and y is the hill coefficient.

This allows the estimation of a time independent potency value (EC50). Based on this value the concentration where a certain percentage (x) of the maximal effect is observed can be calculated by the following equation:


$$EC_{x}=PA_{x}=EC_{50}*{\left(\frac{x}{100-x}\right)}^{\frac{1}{y}}$$

This method was applied to the *in vitro* tumor cell killing and cytokine release data derived from the A375 cell line co-cultured with PBMCs and across the various tumor organoids based on patient derived material from different cancer types.

We then used the above assessments for the projection of the human dose range, covering the FIH, PAD and ATD. First, for the MABEL approach defining the FIH dose, the PA30 of the most sensitive readout, in this case tumor cell killing in the A375 cell line was selected. Using the projected human PK parameters, an intravenous dose was calculated, which would lead to a corresponding Cmax, matching the *in vitro* PA30. This approach is in line with the Guidance for Industry on Bispecific Antibody development as documented by Saber et al. (29). The Cmax and corresponding dose was calculated using the simple equation of:


$$C_{max}=\frac{Dose}{Vc}$$

The PAD was calculated using the same approach, but this time targeting the EC50 for tumor cell killing in the less sensitive ovarian carcinoma tumor organoid as this was assumed to better predict for the less sensitive patient population. Additionally, the ATD range was predicted. Doses were derived where the resulting average concentration at steady state (C_avg,ss_) would match the range of EC90 values for tumor cell killing in the various tumor organoids (esophageal, head and neck, and ovarian cancer). The doses to achieve the corresponding C_avg,ss_ were calculated as follows:

$C_{avg_{ss}}=Dose\frac{Dose}{Cl*\;\tau }$,where Cl is the projected human clearance and τ is the dosing interval.

Taken together those different approaches allowed us to define a dose range, starting from a safe starting dose but then also covering anticipated effects in several less sensitive tumor cell types.

## 3 Results

### 3.1 Preclinical safety

**Figure 2:**
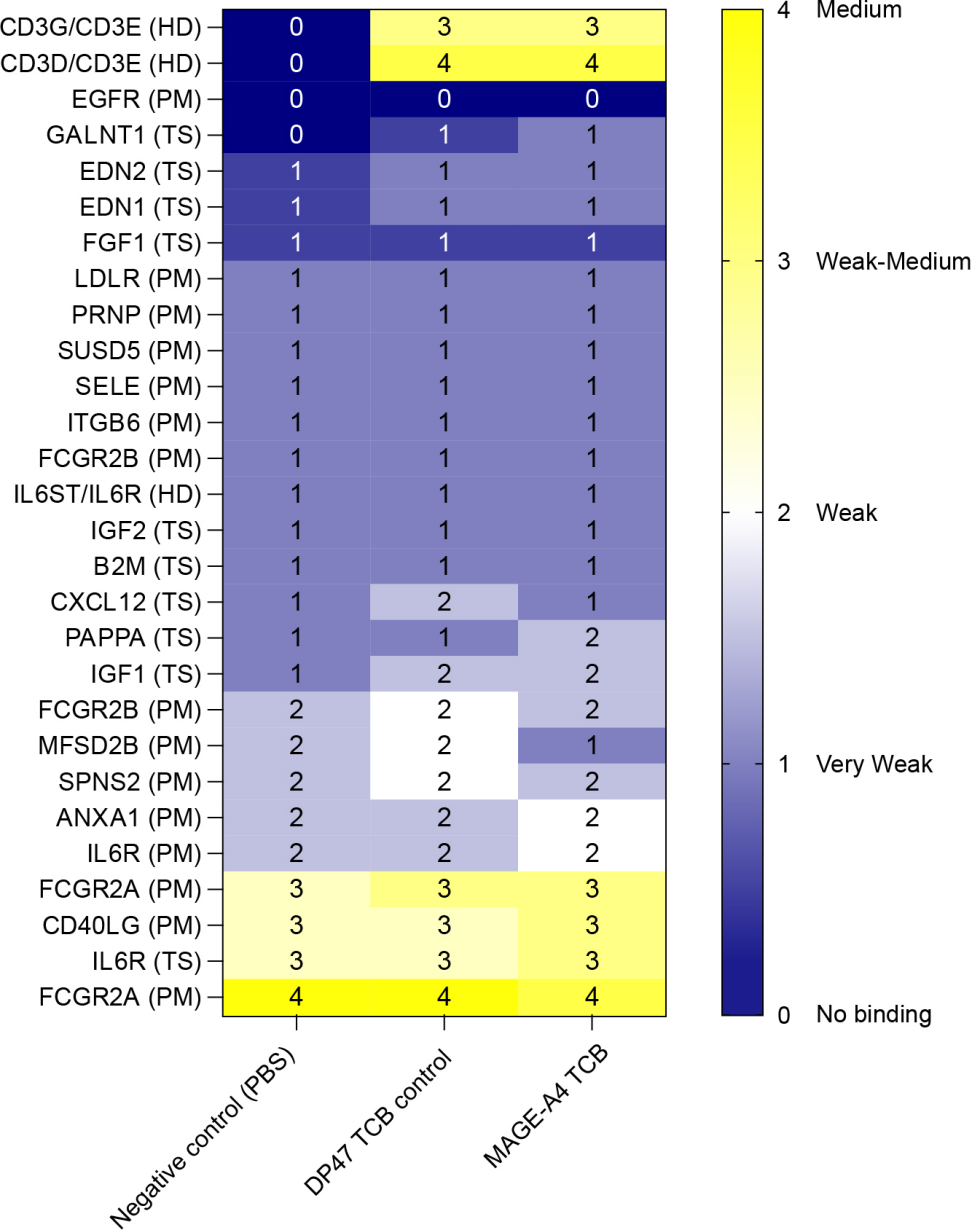
Specificity profiling of MAGE-A4-TCB using the Retrogenix Cell Microarray platform. Assessment of specific off-target binding across a library of 6,101 human plasma membrane and secreted proteins plus 396 heterodimers. The library screen summary is summarized in the table. Binding was observed for the CD3G+CD3E and CD3D+CD3E heterodimers on fixed cell microarrays (n=2), with no binding detected on live cells (n=1), consistent with the requirement for conformational stabilization of the T-cell receptor complex. No specific off-target interactions were identified beyond the primary targets.

The nonclinical safety strategy applied to MAGE-A4-TCB leveraged *in vitro* and ex vivo approaches using human healthy cells and tissues. To increase the likelihood of identifying safety risks related to off-target cross-reactivities, several conventional and less conventional approaches were employed. Since MAGE-A4/HLA-A*02:01 complex presentation is mainly restricted to the tumor; the only anticipated on-target/off-tumor toxicities target the reproductive tract. In line with published data (30, 31), the GLP tissue cross-reactivity (TCR) study performed on human HLA-A2-positive tissues showed positive staining of trophoblastic epithelial cells in the placenta and germinal epithelial cells in the testis besides the staining of mononuclear leukocytes in various tissues due to the binding to CD3. Given the oncology indication, the risk for on-target mediated cytotoxicity in the placenta and testis was considered acceptable and therefore not further tested in an *in vitro* setting. Next, a Human Protein Cell Array (Retrogenix Cell Microarray) was used to screen for specific off-target protein binding interactions (32). In the primary library screen of 6,497 human proteins, 27 library interactions were initially identified for MAGE-A4-TCB with intensities ranging from very weak to medium/strong (**Figure 2**). Subsequent confirmatory screens on fixed cell microarrays demonstrated that 24 of these interactions were non-specific, as they were also bound by the negative control (secondary-only) treatment. These non-specific interactions included several Fc gamma receptors (e.g., FCGR2A, FCGR2B), presumably representing Fc-domain-mediated background binding. Following the exclusion of non-specific and non-significant interactions, MAGE-A4-TCB showed significant, specific binding only to its intended primary targets: CD3G + CD3E (weak/medium intensity) and CD3D + CD3E (medium to weak/medium intensity). This binding profile was identical to that observed for the positive control antibody. Aside from the primary target heterodimers, no other specific off-target interactions were identified, indicating high specificity for MAGE-A4-TCB. (**Figure 2**). In addition, a MAGE-A4-TCB ligandome elucidation approach was applied in which MAGE-A4-TCB was used to enrich bound pHLA-A2 complexes (on- and off-target) in healthy human liver, lung and colon organ lysates. Results were published in 2024 and showed no specific enrichment of peptides in these vital organs (33).

An *in vivo* study was also carried out to evaluate the selectivity of the MAGE-A4pMHC-tumor targeting arm in HLA-A*02:01 human transgenic mice using a hemi-surrogate molecule containing the tumor binding arm of MAGE-A4-TCB and a murine CD3ϵ binder. A single treatment of female AAD transgenic mice with MAGE-A4 (57Do3)-muCD3 hemisurrogate TCB at a dose of 1 mg/kg did not result in test item related gross or histopathology changes in the investigated organs.

Additional standard nonclinical activities, i.e. *in vitro* platelet activation assay, and compatibility of clinical formulation with human blood and plasma completed the *in vitro*-based risk identification exercise and revealed no major finding.

**Figure 3:**
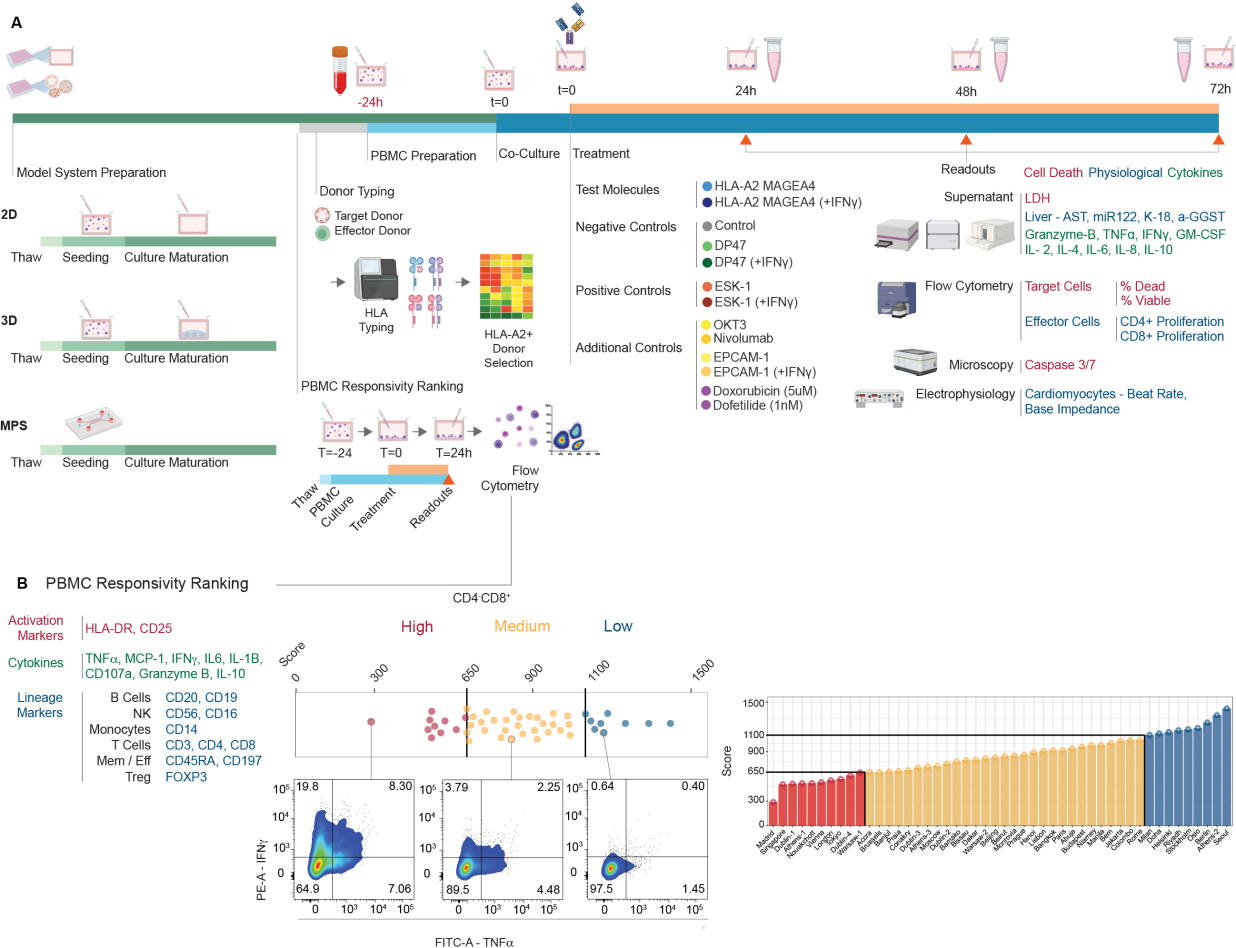
Experimental strategy and effector cell characterization for the NAM-based safety panel. **(A)** General experimental protocol. Target cultures (2D, 3D, or MPS) were established 24 h prior to co-culture. HLA-A*02:01^+^ effector PBMCs were added at a standardized 8:1 E:T ratio (160,000 PBMCs/well). Supernatants and cells were harvested at 24, 48, and 72 h to monitor cytotoxicity, T-cell proliferation, and cytokine release. **(B)** Functional ranking of PBMC donors (n=48). Donors were categorized as low, mid, or high responders based on a composite score of 33 parameters (25 T-cell and 8 leukocyte markers) following 18 h stimulation with a CD20-TCB. Every data point represents an individual donor characterized in technical triplicates.

The risk of targeting vital organs was further evaluated using human *in vitro* 2D and 3D systems derived from human healthy organs in co-culture with autologous or allogeneic human PBMCs in the presence of MAGE-A4-TCB, to identify whether T-cell mediated cell killing is observed. On- or off-target activity was assessed by monitoring the concomitant effects on lysis of target cells, cytokine release and T-cell proliferation (**Figure 3A**). A set of positive controls and negative controls were rigorously chosen: at minimum, a non-targeting TCB control antibody DP47 (with the same antibody backbone as MAGE-A4-TCB) and ESK1, a well characterized TCR-like T cell bispecific antibody known to unselectively target the RMFPNAPYL (RMF) peptide derived from the intracellular tumor antigen Wilms tumor protein (WT1) in the context of HLA-A*02. In addition, whenever possible, certain immune engagers with known toxic effects (Nivolumab, OKT3, EPCAM-TCB) or standard toxicants (Doxorubicin, Dofetilide) were added. Readouts used included a target cell death measurement (fluorescent caspase 3/7, LDH release), cytokine release measurement by multiplex in culture supernatants, CD4+ and CD8+ T cell proliferation and in certain assays physiological parameters such as liver markers (AST, K-18, miR122, a-GGST) or cardiomyocytes beating function.

The clinical candidates were tested on at least six co-cultures, using three PBMC donors and between two to nine primary human cell/tissue donors. The same three PBMC donors were used for all the *in vitro*/ex vivo testing using human cells/tissues (except for the pancreatic islet microtissues which required HLA matched PBMCs), and included a moderately-responding PBMC donor and two highly responding donors. PBMC donors were indeed preliminary ranked as low, mid and high responder based on their response to CD20-TCB as measured by a 33 multiparameter flow cytometry analysis (**Figure 3B**).

**Figure 4:**
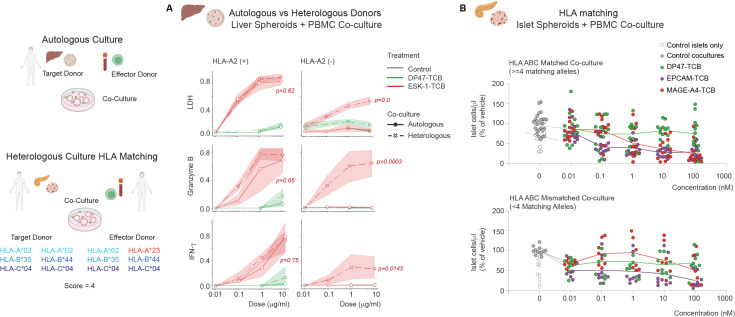
Evaluation of HLA mismatch impact on co-culture sensitivity and background activation. **(A)** Liver spheroid co-cultures comparing autologous and allogeneic (HLA-A*02 matched) effector donors. Data are presented as mean ± SD as shaded area of technical replicates (n=3) from 3 representative donor pairs (N = 3) for each HLA-A*02 background. Statistical significance relative of the autologous cultures compared to corresponding allogenic cultures two-way ANOVA with Tukey’s *post-hoc* test, p values are indicated for the overall comparison between autologous versus allogeneic data. **(B)** Pancreatic islet spheroid co-cultures comparing HLA-matched (4/6 alleles) and HLA-mismatched conditions. Data illustrate the increased signal-to-noise ratio in HLA-matched systems compared to the elevated background activation observed in allogeneic co-cultures. Data are presented as mean of N = 5 donors for the matched dataset and N = 4 donors for the unmatched dataset run in technical replicates (n=2).

**Figure 5:**
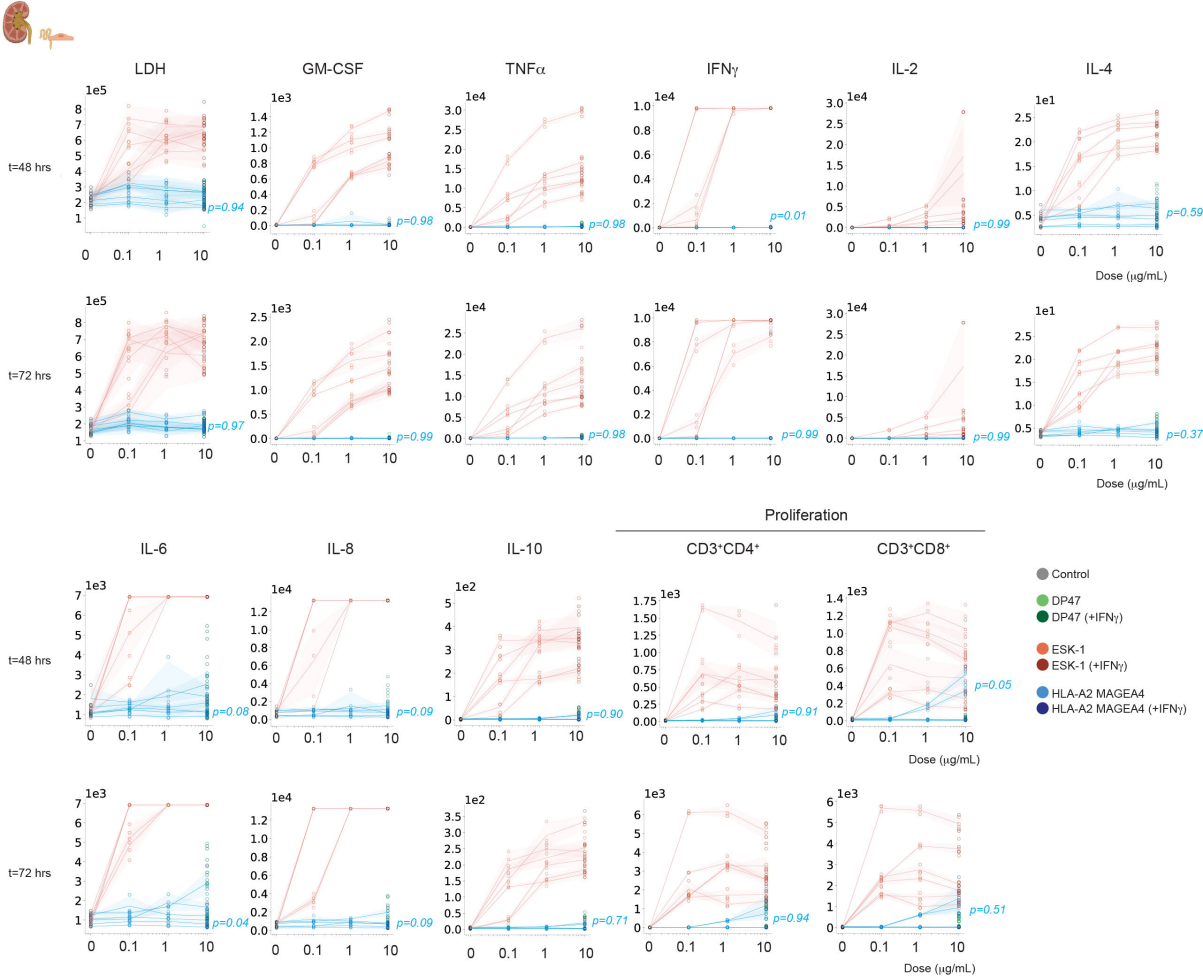
Safety assessment of MAGE-A4-TCB in primary renal proximal tubule epithelial cells (RPTEC). Dose-response behavior for cytotoxicity and T-cell proliferation at 48 h (top) and 72 h (bottom). Each individual line represents the mean of one effector-target donor pair (N = 3 independent donor pairs), with data points showing individual technical replicates (n=3 per donor pair). Shaded areas represent the SD. Statistical significance relative to the untreated control was determined by one-way ANOVA with Tukey’s *post-hoc* test; no significant reactivity was observed for MAGE-A4-TCB (Stimulation Index (SI) < 1.8 for lysis; SI < 5 for proliferation) in comparison with DP47-TCB control assuming significant difference at alpha<0.05 besides IL-6 release at 72h.

Due to the logistical challenges associated with sourcing autologous PBMCs for all co-culture pairs, the *in vitro* testing was performed with allogeneic PBMCs. The impact of that mismatch was, however, evaluated in a set of different cultures (**Figure 4**). Liver spheroid testing was done in autologous versus allogeneic conditions thereby revealing a stronger signal caused by heterologous co-cultures resulting in lower EC50s under allogeneic conditions (**Figure 4A**). Additionally Pancreatic islet spheroid testing was performed in HLA mismatched versus HLA matched donors which highlighted a general background inflammation and target cell killing under mismatch conditions (**Figure 4B**). A representative example of the data collected for all human cells/tissues tested is shown in **Figure 5** with 3 donor pairs of renal proximal tubule epithelial cells.

A total of 21 cellular systems were evaluated and focused on vital organs such as the brain, liver, heart, kidney, lung, intestine, vasculature, pancreas, skin, blood and bone marrow. The choice of the cells to represent an organ was made based on their abundance in that particular organ and their structural/functional relevance. Most cells were of primary origin (keratinocytes, melanocytes, bone marrow mononuclear cells, microvascular endothelial cells, bronchial epithelial cells) while others were iPSC derived (cardiomyocytes, astrocytes). Static 2D co-culture systems with allogeneic healthy PBMCs are considered to represent the worst-case scenario with regard to exposure of target and effector cells to the drug, and the direct contact of the effector with the target cells. In most cases that format was prioritized over 3D models unless the cell type of interest was not available or maintained in its viable and functional form in 2D (liver spheroids, pancreatic islet microtissues, duodenum and colon organoids, alveolar epithelium chip). IFNγ pretreatment was tested in parallel to baseline conditions to model inflammation that may arise in a cancer patient and trigger HLA upregulation.

**Figure 6:**
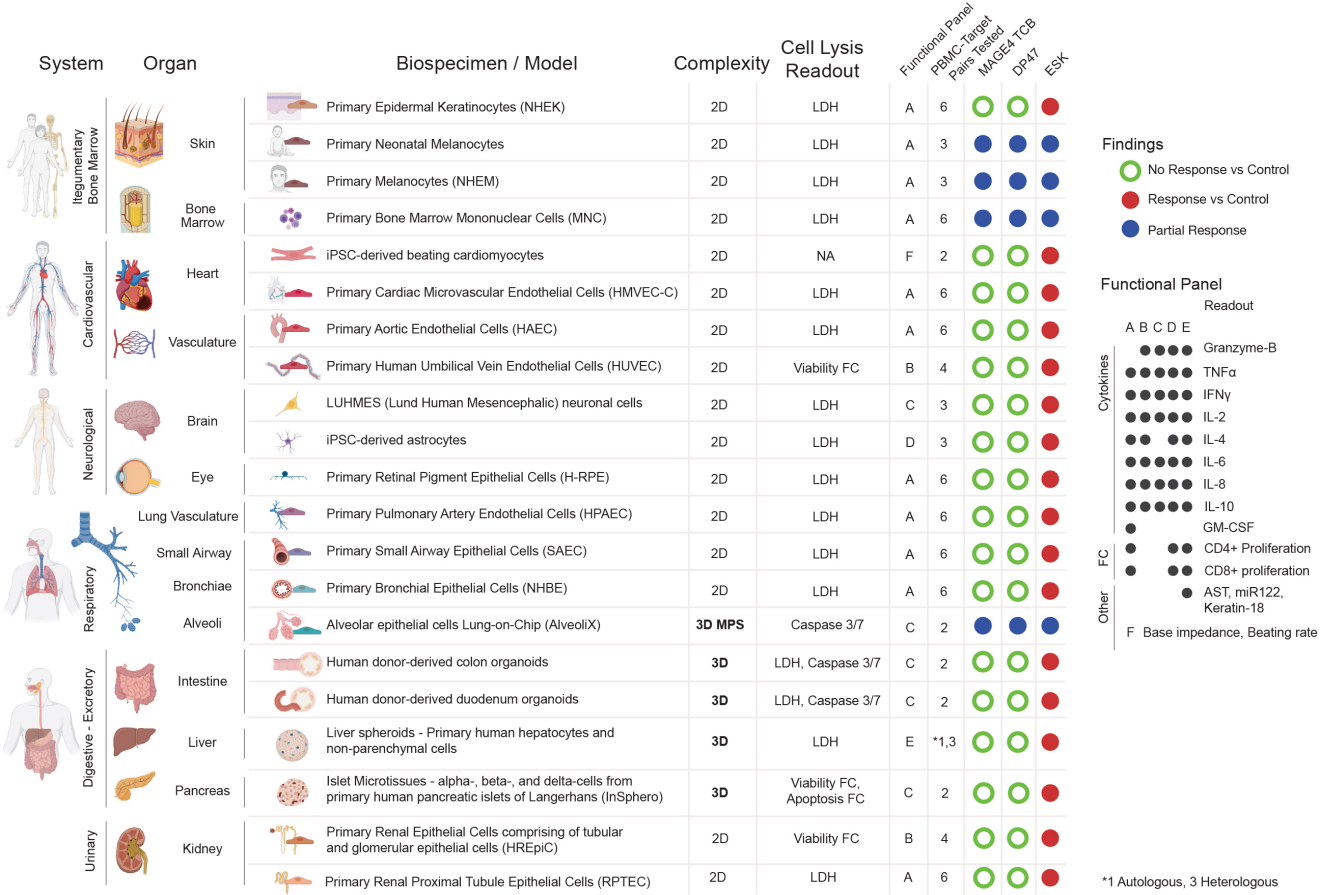
Comprehensive *in vitro* safety assessment across 21 human cellular systems. Panel of *in vitro* models used, categorized by their corresponding physiological system and organ including model dimensionality (2D, 3D, or MPS). Primary cell lysis readouts include Lactate Dehydrogenase (LDH) for cell death, Caspase 3/7 probe for apoptosis, and viability/apoptosis by Flow Cytometry (FC). Functional panels include 8-plex cytokine release (Luminex), T-cell proliferation (EdU), and organ-specific physiological markers (e.g., AST, miR-122, Keratin-18, TEER, beating rate). The combination of readouts for each model is depicted as measured within each designated panel on the right (A–E). In addition, the number of PBMC-target pairs tested is shown for every model. Colored circles represent the assessment relative to controls: Green (no reactivity), Blue (reactivity comparable to the non-targeted DP47-TCB control), and Red (positive response). The number of PBMC-target donor pairs tested is indicated for each system. All detailed results can be found in supplementary data package 1.

No significant effects were observed upon treatment with MAGE-A4-TCB (without and with IFN-γ) when compared with the non-targeted DP47-TCB, while the positive control ESK-1–TCB triggered significant target cell lysis, cytokine release and T-cell activation/proliferation in all 2D/3D *in vitro* systems evaluated, from the lowest concentrations and the earliest time point evaluated. The results of the safety testing for MAGE-A4-TCB are summarized in **Figure 6** and all detailed results are provided in **Supplementary Figures**.

The *in vitro* testing of MAGE-A4-TCB on human primary cells/tissues in co-culture with PBMCs provided evidence for the absence of off-target-reactivity in human *in vitro* systems, as no significant effects were observed on cell lysis, T-cell activation/proliferation or cytokine release in comparison with the non-targeted TCB control. Beyond the on-target/off tumor related risks on placenta and testis, no additional safety risk related to off-target-reactivities was identified for MAGE-A4-TCB. Note that the response in melanocytes, bone marrow and alveolar cells was equally pronounced with controls and MAGE-A4 and therefore upfront not considered relevant/useful for initial safety assessment.

**Figure 7:**
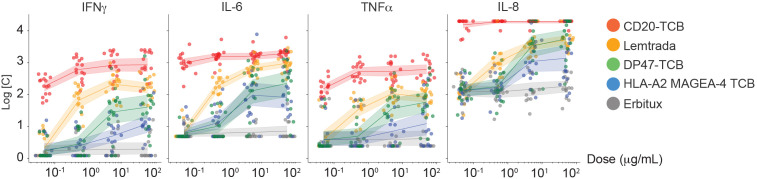
Risk assessment of target-independent cytokine release in human whole blood. Serum levels of TNF-α, IL-6, IL-8, and IFN-γ following 24 h incubation with MAGE-A4-TCB or controls (n=30 donors; 14 HLA-A02^+^ and 16 HLA-A02^-^). Each data point represents an individual donor. Solid lines indicate the mean of the donor population, and shaded areas represent the SD. Positive response thresholds were defined for each donor using a 95% quantile approach relative to the low-risk clinical comparator Erbitux. Differences in cytokine induction between HLA-A*02 positive and negative donor cohorts were evaluated using a two-way ANOVA followed by Sidak’s multiple comparison test. No statistically significant differences (p < 0.05) were observed between the two cohorts, and MAGE-A4-TCB values remained below the calculated significance thresholds across all concentrations tested.

In a human whole blood cell assay, MAGE-A4-TCB induced a dose-dependent release of TNF-α, IL-6, IL-8, and IFN-γ between 0.5 and 50 nM (**Figure 7**). Cytokine levels were in the same range for the non-targeted control DP47-TCB, but considerably lower than cytokine levels induced by alemtuzumab or glofitamab. In addition, no statistically significant differences were observed between HLA-A*02 positive and negative donors at any cytokine/concentration condition, which excludes non-specific binding of MAGE-A4-TCB to HLA-A*02, either in the absence of peptide or in the presence of an off-target peptide presented by HLA-A*02 receptors on the surface of human blood cells.

In conclusion, the main safety risk was anticipated to be related to cytokine release, either due to MAGE-A4-TCB-mediated target-independent T-cell activation or as a result of MAGE-A4-TCB-induced lysis of MAGE-A4/HLA-A*02:01 presenting cancer cells.

### 3.2 Relevant tumor models to predict the anticipated MAGE-A4-TCB effects in patients with solid tumors

**Figure 8:**
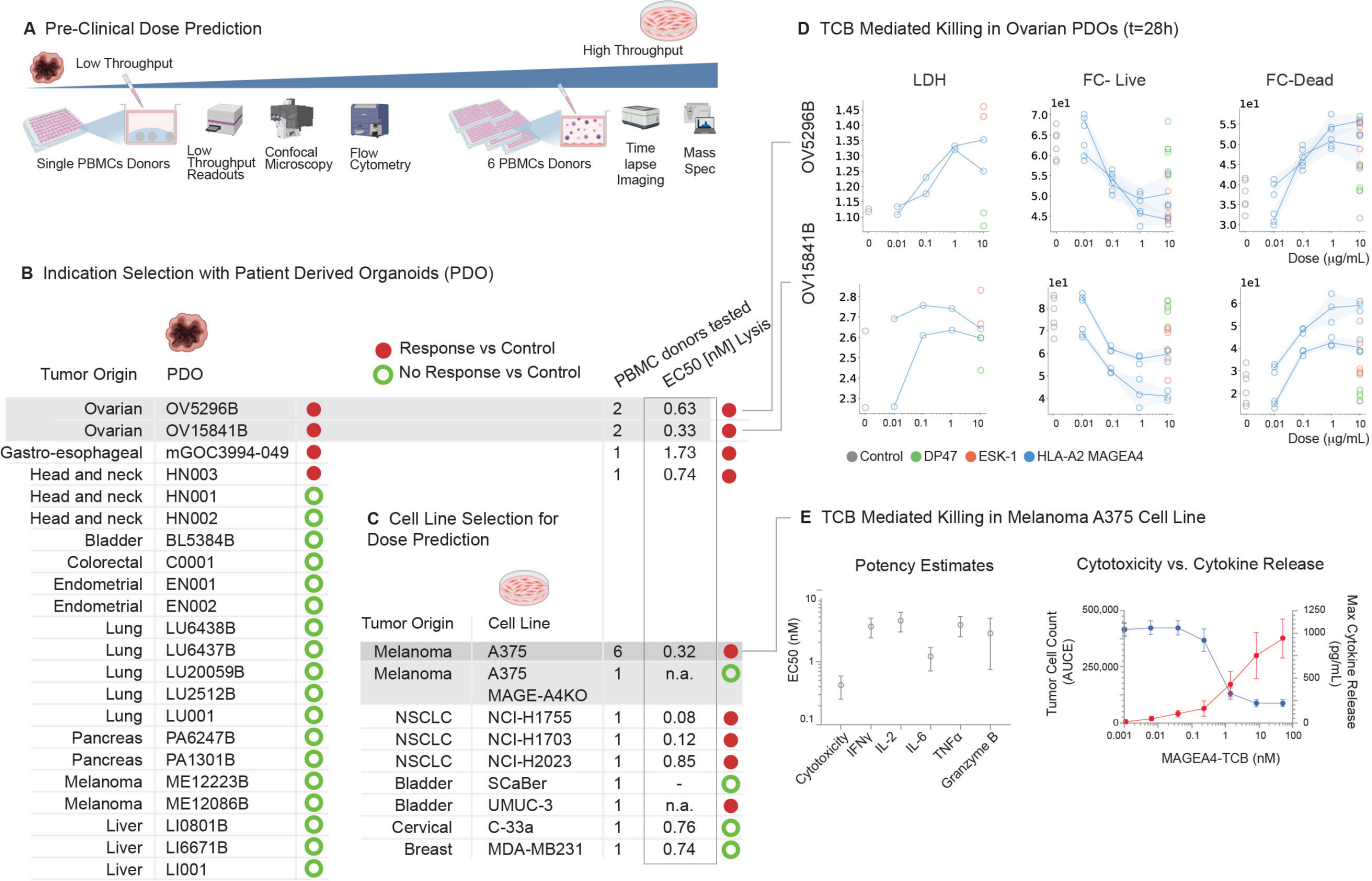
Rationale for tumor model selection and pharmacokinetic-pharmacodynamic (PKPD) profiling **(A)** PDOs provide a more physiologically relevant model but can only be assessed with low throughput technologies, while cell lines can be screened with high throughput technologies, making them suitable for bulk analysis. **(B)** Out of 22 tested tumor organoids, four responders and 18 non-responders were identified, with ovarian tumor organoids demonstrating the highest sensitivity to MAGE-A4-TCB. **(C)** From the cell lines tested, the A375 melanoma cell line was selected as a representative model, exhibiting comparable cytotoxicity (EC50 = 0.32 nM) to the sensitive ovarian tumor organoids (EC50 = 0.32 – 0.6 nM). **(D)** The TCB Mediated killing of ovarian PDO’s was assessed by LDH secretion, and count of Live and Dead cells though Flow Cytometry (FC). **(E)** PKPD results of A375 tested with 6 different PBMC donors (N = 6). left: Data are shown as mean ± SD of potency estimates from technical triplicates of cytotoxicity and release of IFNg, IL2, IL6, TNFalpha and Granzyme B tested in A375 PBMC co culture. Right: Therapeutic index depicted as the cumulative anti-tumor effect (blue line, mean and SD) and IL 6 release (red line, mean and SD).

MAGE-A4 is expressed across a variety of solid tumors, with particularly high prevalence in adenoid cystic carcinoma, liposarcoma, ovarian carcinoma, non-small cell lung cancer (NSCLC), head and neck squamous cell carcinoma (HNSCC), and esophageal cancer, highlighting its potential as a broadly relevant tumor-associated antigen (20). We have therefore selected tumor organoids derived from a wide range of solid tumors (**Figure 8A**). Out of 22 different tumor organoids tested, 4 responder and 18 non-responder tumor organoids were identified, with ovarian tumor organoids being the most sensitive to MAGE-A4-TCB (**Figures 8B,C**). A specific and robust mass spectrometry (MS)-based assay was developed to quantify MAGE-A4/HLA-A02:01 peptide-MHC (pMHC) complex density and evaluate its relationship with MAGE-A4-TCB pharmacodynamics in Patient Derived Organoids (PDO) as well as relevant cancer cell lines. The assay demonstrated high sensitivity (LLOQ = 0.27 fmol; LLOD = 0.09 fmol), with target expression ranging from 6 to over 1000 copies per cell and results are summarized in **Supplementary Figure 2**. MAGE-A4 mRNA expression and protein expression were also evaluated by RTqPCR and western blotting, respectively. While higher complex density generally correlated with increased potency and efficient tumor cell lysis (TCL) at low pMHC levels, certain cell lines, such as ScaBER and C33-a, remained resistant despite substantial surface expression. Notably, no systematic linear relationship was observed between MAGE-A4 mRNA, HLA-A02:01 surface levels, or pMHC copy number and the overall potency of MAGE-A4-TCB. When comparing potency estimates of tumor lysis to those derived from the cancer cell line panel, the A375 cell line was identified as a relevant cell line with similar potency (EC50) with regards to tumor cytotoxicity (EC50 = 0.32 nM) as compared to the ovarian tumor organoids (EC50 = 0.32 – 0.6 nM) (**Figure 8C**). The A375 cell line was subsequently used to establish the nonclinical PKPD relationship from time-course experiments assessing tumor cell cytotoxicity as well as cytokine release (**Figure 8E**).

### 3.3 PK/PD and human dose range prediction

As summarized in **Table 1**, from all measured *in vitro* readouts in A375, the most sensitive was tumor cell killing followed by IL6 release. Therefore, we used the concentration-effect curve of A375 tumor cell killing, to inform a rather conservative *in vitro* MABEL approach. The *in vitro* PA30 (30% pharmacological activity) derived from tumor cell killing was 0.06 µg/mL. The PA30 for IL6 release was approximately 2x higher than for tumor cell killing (0.14 µg/mL).

**Table 1:** Median PA30 (30% pharmacological activity) based on *in vitro* readouts using the A375 cell line.

*In vitro* readout	A375 median PA30 (µg/mL)
Cytotoxicity	0.06
IL-6	0.14
IFN-γ	0.29
TNF-α	0.29

The hFcRn tg32 mouse PK parameters were estimated based on the SDPK study and the estimated clearances subsequently scaled by allometry to human values. In terms of the volume of distribution, common human values are assumed. The hFcRn mouse- and scaled human parameters are summarized in **Table 2**.

**Table 2:** PK parameters in hFcRn mice projected to human.

PK parameters	hFcRn mice est. (%RSE)	Projected to human*
CL (mL/day)	350 (6.9)	420
Vc (mL)	1475 (29)	3100
CLd (mL/day)	1220 (41)	1510
Vp (mL)	2910 (6.7)	4070

*Fixed to generic human values for volumes, scaled according to material and methods for clearances.

Based on the predicted human PK parameters a human starting dose was derived, which would lead to a human serum Cmax of 0.06 µg/mL, i.e. matching the PA30 of tumor cell killing in the A375 cell line (most sensitive readout/system), resulting in a human starting dose of 0.175 mg.

**Figure 9:**
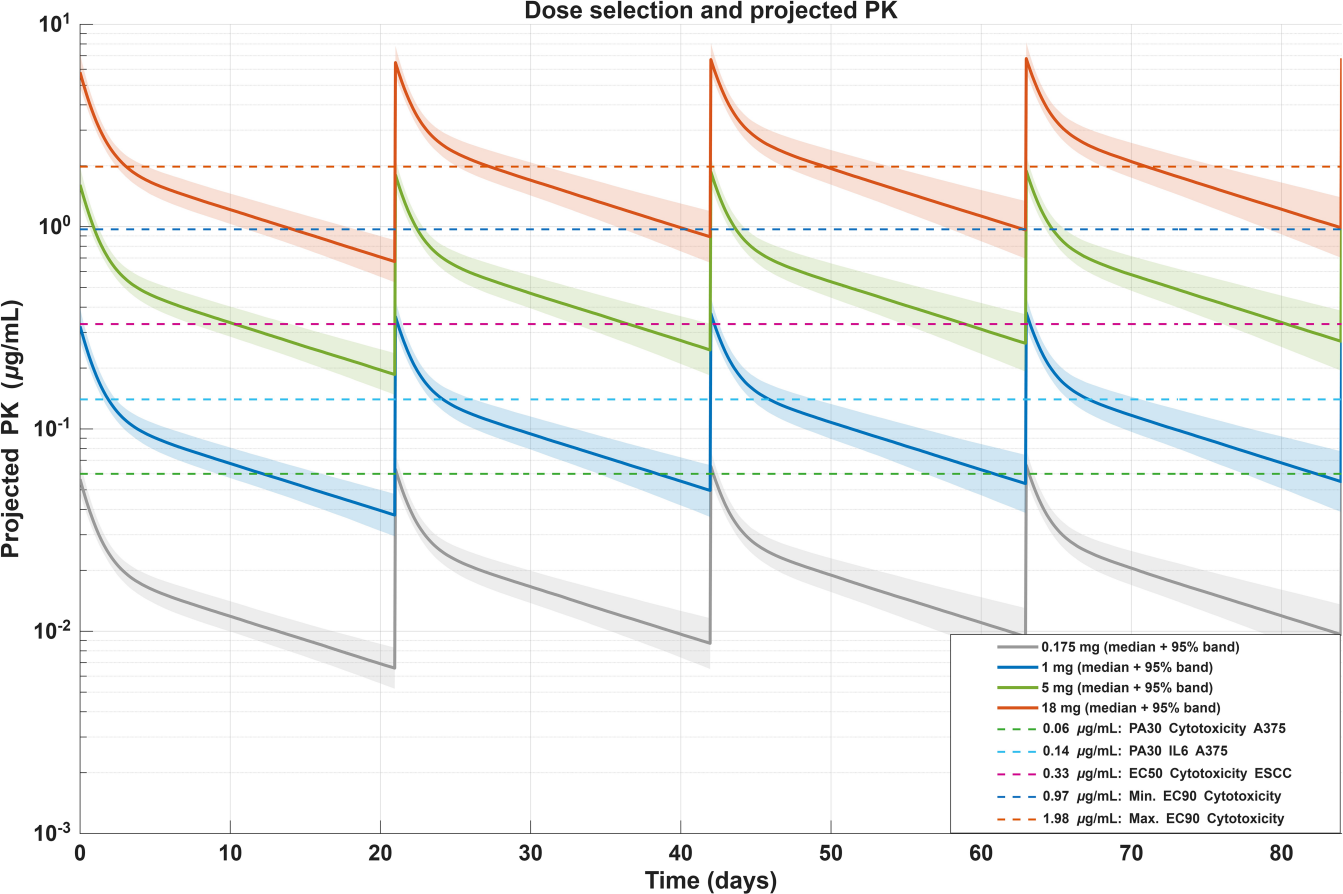
Human pharmacokinetic (PK) projection and dose determination. Simulated human serum concentration-time profiles for MABEL (0.175 mg), PAD (1 mg), and ATD (5–18 mg) dose levels under a Q3W intravenous regimen. Solid curves represent median profiles extrapolated from hFcRn-tg32 mouse PK using linear allometric scaling. Shaded areas illustrate 95% uncertainty bands derived from ±20% sensitivity simulations of clearance and volume of distribution parameters. Dashed horizontal lines indicate targeted *in vitro* activity thresholds (PA30 and EC50 from tumor-killing and cytokine-release assays.

The EC50 in the less sensitive ovarian tumor organoid (OV15841B) was estimated at 0.33 µg/mL and led to the calculation of a pharmacologically active dose (PAD) of 1mg. The anticipated therapeutic dose (ATD)-range of 5 to 18 mg, was based on the concentrations at which 90% tumor cell lysis (EC90 = 0.97 – 1.98 µg/mL) was observed in various tumor organoids, which were assumed to capture the range of patient populations of interest, originating from esophageal, head and neck and ovarian tissues. The doses were derived by matching the expected Cavg,ss in humans at the respective doses to the EC90 range. The corresponding efficacious dose-range in humans was predicted to be 5 to 18 mg assuming a Q3W dosing regimen. To assess the robustness of the projected human PK, a sensitivity analysis was performed in which CL, Vc, CLd and Vp were independently varied by ±20% around their projected values. Concentration–time profiles were simulated for the proposed clinical dose levels and summarized as median profiles with 95% uncertainty bands. These simulations are shown in **Figure 9**.

### 3.4 Clinical experience with MAGE-A4-TCB

**Figure 10:**
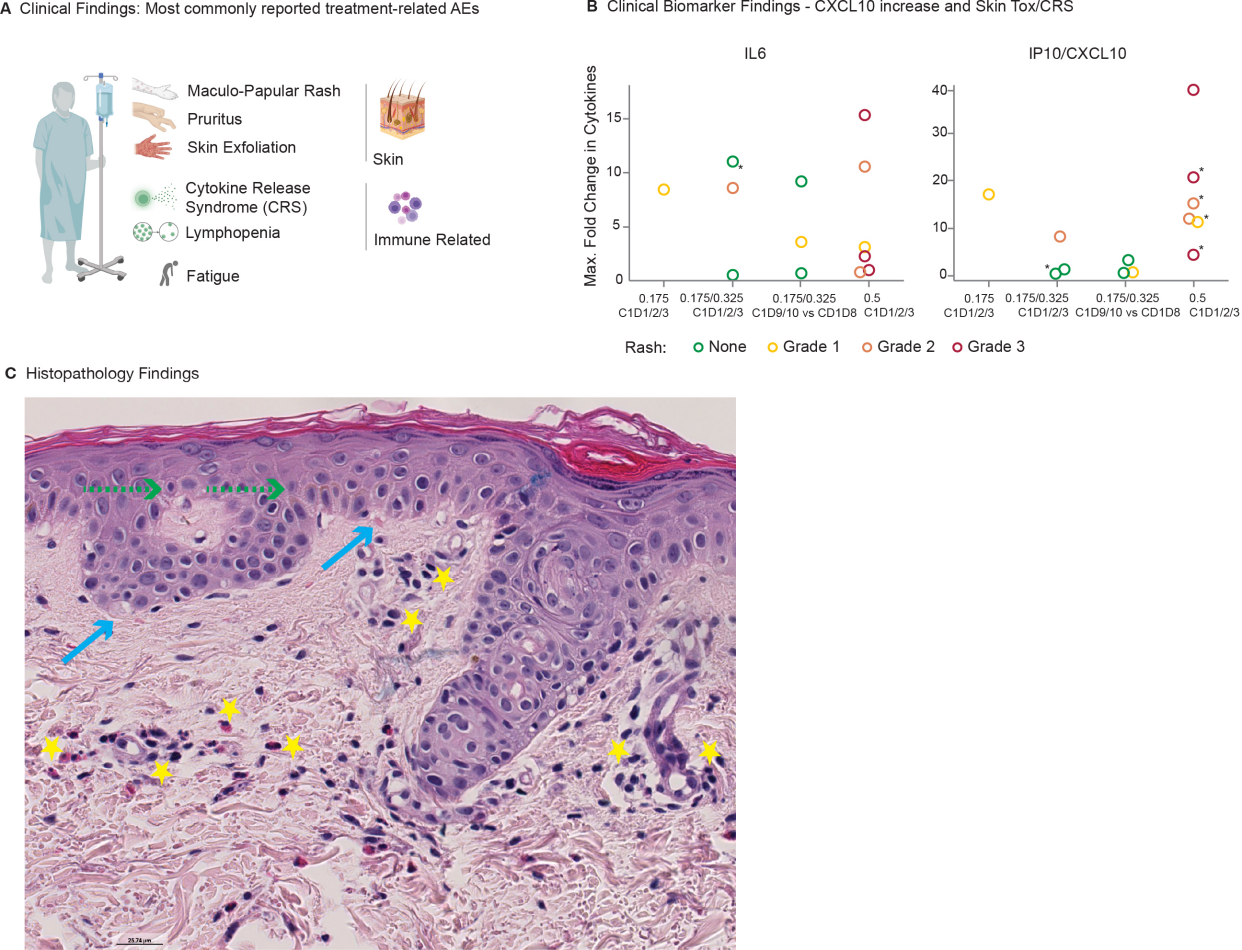
Clinical safety profile and skin adverse events. **(A)** Summary of treatment-related adverse events in Phase I (n=22 patients). **(B)** Maximum fold change in serum CXCL10 (IP-10) levels relative to baseline (C1D1 or C1D8 predose), stratified by the severity of skin rash (Grade 0–3). Each point represents the peak measurement for an individual patient. CXCL10 values for patients 20101, 20102, 20103, and 20104 were above the limit of quantification at baseline, potentially underestimating fold changes. **(C)** Exanthemous drug eruption in a patient-derived on-treatment skin biopsy: histology shows a mild epidermal spongiosis (green arrows), a minimal interface dermatitis (blue arrows) as well as superficial perivascular inflammation (yellow stars) with prominent eosinophils and moderate amount of lymphocytes. Hematoxylin and Eosin (HE), 10x. Bar = 20 µm.

In the Phase I Study (NCT05129280), 22 patients with unresectable and/or metastatic MAGE-A4-positive, solid tumors received at least one dose of MAGE-A4-TCB. The starting/MABEL dose of 0.175 mg IV turned out to be safe. The highest dose tested (following a single step of 0.175 mg) was 1.6 mg, which is still more than 3-fold below the anticipated therapeutic dose. Most commonly reported treatment-related Adverse Events (AEs) (Treatment-Related Adverse Events (TRAEs); ≥10% of patients, mostly Grade 1 and 2) were rash, pruritus, CRS, lymphopenia, fatigue and skin exfoliation, with 73% of patients experiencing treatment related skin and subcutaneous tissue disorders (**Figure 10A**). Most skin related TRAEs were mild or moderate, but 4 of 22 patients (18%) reported Grade 3 rash. All events resolved promptly with supportive care and corticosteroid treatment where appropriate.

Skin findings in patients were associated with CRS and correlated with an increase in IP10/CXCL10 (**Figure 10B**) and a retrospective biomarker analysis revealed that patients with a pro-inflammatory state (higher predose CRP values) at baseline were more susceptible to skin rash. Based on skin biopsies of two affected patients, there was no obvious indication of T-cell mediated killing of epidermal or dermal cells, but a perivascular infiltrate with prominent eosinophils and some lymphocytes (see HE stain in **Figure 10C**). Both patients also experienced concurrent CRS.

**Figure 11:**
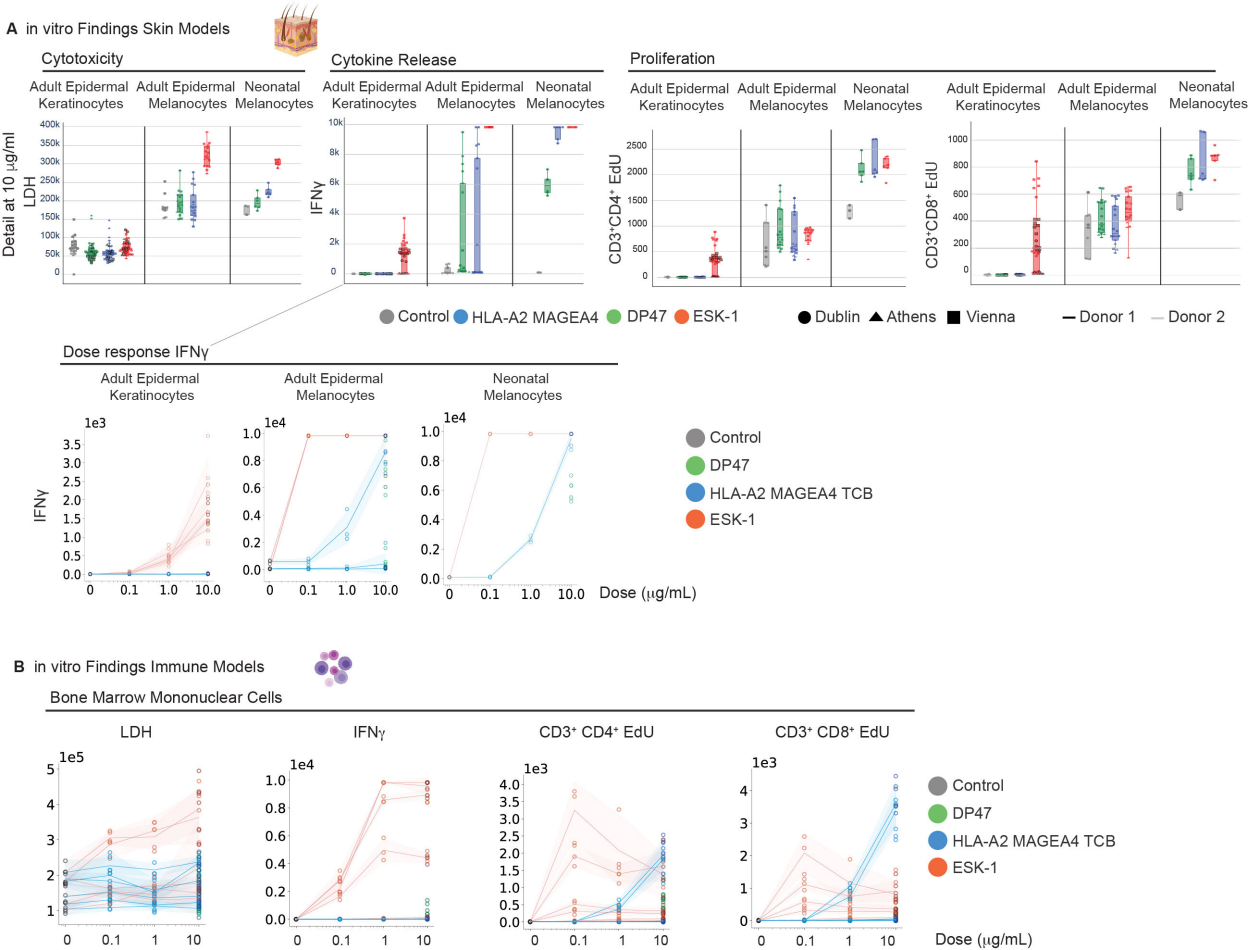
Retrospective *in vitro* investigation of UCHT1-based CD3 binder activity. **(A)** Evaluation of T-cell proliferation and cytokine release in co-cultures with Adult Epidermal Keratinocytes (NHEK), Adult Epidermal Melanocytes (NHEM), and Neonatal Epidermal Melanocytes. Target cells were co-cultured with HLA-A02:01^+^ PBMCs from functionally ranked high-responder donors (N = 2 independent donor pairs per system) as explained previously. Readouts include cytotoxicity (LDH release), T-cell proliferation measured by EdU incorporation in CD3^+^CD4^+^ and CD3^+^CD8^+^ T cell subsets, and pro-inflammatory cytokine secretion (TNF-α, IFN-γ, GM-CSF, IL-2, IL-4, IL-6, IL-8, and IL-10). Data are shown both under basal conditions and following IFN-γ pre-treatment (100 pg/mL) to simulate systemic inflammation and maximize HLA-A02 expression. Each data point represents the mean ± SD of technical triplicates (n=3). **(B)** Dose-response curves of T-cell proliferation in primary bone marrow mononuclear cell co-cultures (N = 2 donors). Each individual line represents the mean of an effector-target pair with shaded areas representing the SD of technical triplicates (n=3 per donor pair). The comparable activation profiles between the clinical candidate and the non-targeted DP47-TCB across these skin cocultures and bone marrow cultures support a target-independent, CD3-mediated mechanism as the primary driver of the skin adverse events observed in the clinic.

The frequency of skin adverse events appeared in contradiction with the results obtained during the *in vitro* assessment of primary keratinocytes and melanocytes. Indeed MAGE-A4-TCB was not associated with elevated target cell killing and cytokines in comparison with the non-targeting DP47-TCB control antibody in those killing assays. However, when looking into the details of the dataset, it appeared that MAGE-A4-TCB and the DP47 TCB control antibody induced cytokine release and proliferation of T cells in co-cultures with neonatal melanocytes (**Figure 11A**), thereby suggesting that the CD3 binder itself may trigger immune activation *in vitro*. MAGE-A4-TCB and DP47 TCB also induced granzyme B and IFNγ release in differentiated alveolar epithelial 3D chip coculture but not in small airway and bronchial epithelial progenitor cell co-culture. Additionally, primary bone marrow mononuclear cell co-culture results demonstrated a similar increase of T cell proliferation with MAGE-A4-TCB and its non-targeting TCB control (**Figure 11B**). These findings in conjunction with the cytokine release observed in the whole blood assay (**Figure 7**) indicate that the UCHT1-based CD3 binder may drive T cell activation in a target-independent manner, clinically manifesting in skin TRAEs.

The clinical study was discontinued, mainly due to a high rate of persistent anti-drug-antibodies (ADAs) with a substantial impact on drug exposure in the majority of patients as well as for strategic reasons. No apparent crosslinking of the TCB leading to late cytokine release was observed. Based on the available data, it was concluded that no safety events could be correlated with the presence of ADAs. Skin findings occurred after the first dose and were therefore not considered ADA related.

## 4 Discussion

This work aimed to develop a translational strategy for FIH studies of a TCR-like T Cell Bispecific (TCB) using an *in vitro*/ex vivo-based safety approach. Due to the elevated risk of off-target toxicity compared to conventional T-cell bispecifics, the testing strategy incorporated over 20 different human cell types. This approach focused on vital organ systems, consistent with prior methodologies used for CAR T cells and ImmTAC (10, 12, 34).

In addition, to ensure regulatory acceptance across all regions, an *in vivo* study in HLA-A*02:01 human transgenic mice was carried out to evaluate the selectivity of the MAGE-A4pMHC-tumor targeting arm. Since 35 to 40% of the human peptidome is shared with the mouse and these peptides can be presented by the huHLA-A2 molecules expressed in these mice, this model was expected to enable the identification of MAGE-A4-TCB off-target reactivities on HLA-A*02:01-presented shared antigens only.

While our findings demonstrate that the starting dose was safe, the small sample size and limited dose range in our clinical trial prevent further validation of the translational relevance of the *in vitro* safety approach. However, the insights gained from the methodology itself are invaluable for refining future *in vitro* based safety assessments of T cell engagers.

### 4.1 Methodological insights

A significant challenge in our approach was the variability in the signal-to-noise ratio across different systems and donor variability, with response magnitudes differing among PBMC donors. To address this, we implemented donor ranking, but variability remains a concern. Autologous or HLA-matched cocultures are recommended, especially for confirming findings from allogeneic models (35, 36). Despite these efforts, it is clear that an *in vitro*-based strategy cannot capture the full diversity of human immune responses and potential target cells (up to 200 different cell types in the human body). The need for standardization across different platforms, as well as practical limitations like material availability, further complicates this effort. Miniaturizing and automating culture systems would help mitigate these challenges.

### 4.2 Cellular context and controls

Primary and iPSC-derived cells were evaluated in static 2D co-cultures with allogeneic PBMCs to establish a worst-case scenario for drug exposure and direct effector-target interaction. This high-access format was prioritized for safety screening, while complex 3D models like organoids or microfluidic chips were reserved for tissues where maintaining differentiated functional profiles required more physiological architecture. These selections ensure that the assessment of MAGE-A4-TCB accounts for both maximum potential reactivity and tissue-specific protein expression. The cell type used in the *in vitro* system plays a critical role in safety testing. We found that more stem-like cells, such as expanded intestinal organoids, led to minimal allogenicity, whereas differentiated cells like alveolar epithelial cells and melanocytes triggered more immune responsiveness. This highlights the importance of selecting the right cell models, as stem-like cells may not fully capture the protein expression profile of a fully differentiated cell, including expression of HLA molecules (37, 38). 3D culture systems offer a more physiologically relevant environment that better mimics tissue architecture and immune interactions (39–41). However, complexity must be balanced with practicality for high-throughput screening.

We also identified the importance of proper controls in those *in vitro* assays. Negative controls (CD3- and tumor antigen-negative) and positive controls (ensuring constant detection of target cells, ideally one in a generic fashion and another in a tissue specific fashion) would be desired in interpreting results accurately. In addition to the non-targeting TCB control, an IgG containing the MAGE-A4-TCB tumor binding arm with a different CD3 binding arm of low affinity or an active Fc domain would have been helpful to discern on/off-target effects from non-specific CD3-mediated effects. Furthermore, running tumor models alongside normal tissue models is crucial to evaluate the potential therapeutic window of the tested drug candidate (42, 43). It also enabled the determination of the starting dose. To further focus on patient-centric approaches, it could be beneficial to utilize normal cells and PBMCs obtained directly from cancer patients.

### 4.3 Cytokine response and inflammatory toxicity in barrier organs

Unexpectedly, skin adverse events were the most commonly reported events in the FIH study which may mirror immune-related adverse events seen with other immune-stimulators like T cell engagers, CAR-Ts, cytokine-based therapies or checkpoint inhibitors (44, 45). This effect might be driven by the CD3 arm of the molecule, potentially inducing an inflammatory response through cytokine release, especially in patients with a systemic pro-inflammatory profile, as reflected by higher predose CRP values found in patients with skin events of our trial similarly to previously reported studies (46, 47). Cytokine release in a whole blood assay and cytokine release and proliferation of T cells in coculture killing assays of bone marrow cells, alveolar epithelial cells, and melanocytes, with both, MAGE-A4 -TCB and DP47-TCB support this hypothesis. However, at the current stage of development, it remains challenging to distinguish whether the observed clinical toxicity is purely target-independent (CD3-mediated) or an additive effect involving low-level MAGE-A4 or off-target binding. It is important to note that immunopeptidomics (ligandome) and flow-cytometry-based binding assays failed so far to detect MAGE-A4 peptide presentation on primary human melanocytes or in healthy skin tissues. This suggests that the clinical skin events observed are likely driven by the high sensitivity of the skin’s immune microenvironment to low-level, target-independent CD3 engagement. While current NAMs may have identified this risk signal, future models incorporating resident immune populations and localized barrier immunity may better calibrate the translation of target-independent activation to clinical adverse event frequency.

The skin, in particular, contains a substantial population of α***β*** tissue-resident memory T cells (TRM), which are key players in local immune responses (48–50). These TRM cells are known to contribute to the skin’s defense against pathogens but may also drive inflammatory responses when activated inappropriately, as observed in autoimmune diseases (51). Notably, in our clinical study, we observed an increase in IP-10 (CXCL10) levels, a cytokine commonly associated with skin inflammation. This observation aligns with findings in vitiligo patients, where IFN-γ signaling via the CXCL10 pathway is implicated in the development of skin lesions (52).

An additional layer of complexity in the skin’s immune response is contributed by ***γ**δ* T cells, a distinct lineage of T lymphocytes highly enriched in barrier tissues (53). Like α***β***, a significant fraction of these ***γ**δ* T cells exhibits a tissue-resident phenotype in the skin, acting as rapid, early responders to tissue stress or damage. It is plausible that the observed skin adverse events are, in part, driven by the molecule’s mechanism of action. Specifically, the non-MHC-restricted T-cell engaging component, which targets CD3, may inadvertently engage the CD3 TCR complex on these skin-resident ***γ**δ* T cells (54). Given the relatively higher abundance of ***γ**δ* T cells in the skin compared to some other tissues, and their potent capacity for rapid IFN**γ** and inflammatory cytokine production, this engagement could lead to their non-specific activation and subsequent release of pro-inflammatory cytokines, driving the observed increase in IP-10 and clinical skin inflammation.

In general, the role of barrier organs, which contain tissue-resident T cells such as α***β*** TRM and ***γ**δ* T cells, is crucial in predicting toxicity of immunotherapies. As highlighted by Recaldin et al. (55), these organs greatly contribute to immune responses, and their inclusion in *in vitro* models may improve the accuracy of safety predictions. Future studies incorporating these critical tissue resident immune cells will provide a more realistic framework for evaluating TCB toxicity (42, 56).

In 2024 the FDA approved a biologics license application (BLA) for the investigational T-cell therapy afamitresgene autoleucel (afami-cel,Tecelra^®^), targeting MAGE- A4 antigen, in the management of advanced synovial sarcoma (57, 58). Afami-cel is the first engineered T-cell therapy on the market for a solid tumor, validating the target in the context of cell-based therapy. In a Phase 1 study of afami-cel 9 of 32 patients had skin rashes possibly related to afami-cel (18). Skin rashes were reversible with supportive care and/or topical corticosteroids with clinical labels currently citing only localized alopecia. The mechanisms behind may or may not be comparable to our observations with MAGE-A4-TCB.

### 4.4 PK/PD

The hFcRn transgenic mouse model assesses non-specific antibody clearance but lacks MAGE-A4 cross-reactivity, thus only characterizing the non-specific linear PK process. While conservative for safety by predicting maximum exposure without target-mediated drug disposition (TMDD), it risks underexposure if TMDD occurs. Predicting TMDD quantitatively is challenging without cross-reactive species or clinical data. Future developments in NAMs, combining mathematical models and machine learning, *in vitro* derived parameters, and in silico predictions, show promise (59). *In vitro* systems also assess non-specific clearance (60–62) and have been shown to achieve classification of therapeutic antibodies based on clearance (63) as well as ranking and mechanistic assessment. However, further development and validation is required for quantitative human PK predictions. As such, for now, the hFcRn tg32 mice will likely remain the primary method for human PK prediction. We scaled clearances based on huFcRn tg32 mice, using common human distribution volumes, which is a good approach when no target-mediated drug disposition is expected (26, 27).

In a linear PK framework, the MABEL approach, which is driven by C_max_, is primarily sensitive to uncertainty in the central volume of distribution, whereas the ATD approach, which is driven by C_avg,ss_, is primarily sensitive to uncertainty in clearance. Sensitivity simulations/calculations confirm that proportional variability in these parameters results in proportional changes in exposure and calculated MABEL or ATD respectively.

The selection of PA30 as a MABEL dose aligns with FDA bispecific antibody guidance (29). Determining the MABEL dose involves selecting the most relevant readout and system. While using the most sensitive readout/system is safe, it risks starting at low doses and requiring multiple escalations for efficacy. System sensitivity is influenced by cell line, target expression, effector:tumor cell ratio, donor, readout, and measurement timepoints. Dose selection also considers targeting EC20, EC30, or EC50 by matching projected human C_max_, C_avg_, or C_trough_, all affecting the calculated dose. The goal is to balance a safe starting dose with a translationally relevant system and readout. We assessed tumor organoids for drug potency, then selected the A375 cell line for preclinical testing, matching organoid potency. The PA30 of the most sensitive readout (tumor cell killing) in A375 cells defined the MABEL dose, while organoid readouts projected the therapeutic dose range (PAD and ATD). Tumor organoids could soon offer a way to predict clinical drug responses (64).

### 4.5 The future of *in vitro* safety assessment

While this study was driven by a lack of cross-reactivity in animal models, we envision a safety assessment based on alternative or animal-free methods becoming a future standard for most large molecules. To finally achieve this, substantial industry-wide efforts are required to validate and refine these methodologies. Our study offers a strong foundation for these future advancements, and the lessons learned will be critical in improving safety testing methodologies as now encouraged by regulatory agencies such as EMA and FDA (65, 66).

In conclusion, our findings demonstrate the potential of *in vitro* models for safety evaluation, though challenges remain in model complexity, consistency, integration with other tools and accurate prediction of irAEs. Nevertheless, the insights collected from this study are expected to inform future strategies for optimizing safety assessments of T-cell bispecific antibodies and similar immunotherapies.

## Data Availability

The original contributions presented in the study are included in the article/**Supplementary Material**. Further inquiries can be directed to the corresponding authors.

## Supplementary material

Supplementary Figure 2 **(A)** Quantification of MAGE-A4 expression in tumor models. (Left) Density of MAGE-A4/HLA-A*02:01 complexes quantified by targeted liquid chromatography-tandem mass spectrometry (LC-MS/MS) (Right) Relative MAGEA4 mRNA levels quantified by real-time qPCR, expressed as a percentage of the expression levels measured in the A375 reference cell line. Data represent the mean SD of technical replicates (n=3-4) **(B)** Western blot analysis of total MAGE-A4 protein levels in extracts from patient-derived organoid (PDO) lines using a rabbit monoclonal anti-MAGE-A4 antibody. Detection of GAPDH served as a loading control. **(C)** Correlation of target expression levels with MAGE-A4 TCB cytotoxic potency (EC50). Higher cytotoxic potency (lower EC50 values) generally correlates with higher complex densities. While efficient tumor cell lysis was observed at low complex levels in the A375 melanoma cell line (~19 copies/cell), responding PDO lines (e.g., ovarian) consistently demonstrated densities > 40 copies per cell.

## References

[1] Carlino MS Larkin J Long GV . Immune checkpoint inhibitors in melanoma. Lancet. (2021) 398:1002–14. doi: 10.1016/s0140-6736(21)01206-x. PMID: 34509219

[2] Yoo S-K Fitzgerald CW Cho BA Fitzgerald BG Han C Koh ES . Prediction of checkpoint inhibitor immunotherapy efficacy for cancer using routine blood tests and clinical data. Nat Med. (2025) 31:869–80. doi: 10.1038/s41591-024-03398-5. PMID: 39762425 PMC11922749

[3] Middelburg J Kemper K Engelberts P Labrijn AF Schuurman J van Hall T . Overcoming challenges for CD3-bispecific antibody therapy in solid tumors. Cancers Bsl. (2021) 13:287. doi: 10.3390/cancers13020287. PMID: 33466732 PMC7829968

[4] Klebanoff CA Chandran SS Baker BM Quezada SA Ribas A . T cell receptor therapeutics: immunological targeting of the intracellular cancer proteome. Nat Rev Drug Discov. (2023) 22:996–1017. doi: 10.1038/s41573-023-00809-z. PMID: 37891435 PMC10947610

[5] Kingwell K . T cell receptor therapeutics hit the immuno-oncology stage. Nat Rev Drug Discov. (2022) 21:321–3. doi: 10.1038/d41573-022-00073-7. PMID: 35440812

[6] Holland CJ Crean RM Pentier JM de Wet B Lloyd A Srikannathasan V . Specificity of bispecific T cell receptors and antibodies targeting peptide-HLA. J Clin Invest. (2020) 130:2673–88. doi: 10.1172/jci130562. PMID: 32310221 PMC7190993

[7] Middleton MR McAlpine C Woodcock VK Corrie P Infante JR Steven NM . Tebentafusp, a TCR/anti-CD3 bispecific fusion protein targeting gp100, potently activated antitumor immune responses in patients with metastatic melanoma. Clin Cancer Res. (2020) 26:5869–78. doi: 10.1158/1078-0432.ccr-20-1247. PMID: 32816891 PMC9210997

[8] Nathan P Hassel JC Rutkowski P Baurain J-F Butler MO Schlaak M . Overall survival benefit with tebentafusp in metastatic uveal melanoma. N Engl J Med. (2021) 385:1196–206. doi: 10.1056/nejmoa2103485. PMID: 34551229

[9] Jones HF Molvi Z Klatt MG Dao T Scheinberg DA . Empirical and rational design of T cell receptor-based immunotherapies. Front Immunol. (2020) 11:585385. doi: 10.3389/fimmu.2020.585385. PMID: 33569049 PMC7868419

[10] Harper J Adams KJ Bossi G Wright DE Stacey AR Bedke N . An approved *in vitro* approach to preclinical safety and efficacy evaluation of engineered T cell receptor anti-CD3 bispecific (ImmTAC) molecules. PloS One. (2018) 13:e0205491. doi: 10.21956/openreseurope.14914.r27773 30321203 PMC6188753

[11] Chen X Kamperschroer C Wong G Xuan D . A modeling framework to characterize cytokine release upon T-cell-engaging bispecific antibody treatment: Methodology and opportunities. Clin Transl Sci. (2019) 12:600–8. doi: 10.1111/cts.12662. PMID: 31268236 PMC6853151

[12] Cameron BJ Gerry AB Dukes J Harper JV Kannan V Bianchi FC . Identification of a Titin-derived HLA-A1-presented peptide as a cross-reactive target for engineered MAGE A3-directed T cells. Sci Transl Med. (2013) 5:197ra103. doi: 10.1126/scitranslmed.3006034. PMID: 23926201 PMC6002776

[13] Linette GP Stadtmauer EA Maus MV Rapoport AP Levine BL Emery L . Cardiovascular toxicity and titin cross-reactivity of affinity-enhanced T cells in myeloma and melanoma. Blood. (2013) 122:863–71. doi: 10.1182/blood-2013-03-490565. PMID: 23770775 PMC3743463

[14] van den Berg JH Gomez-Eerland R van de Wiel B Hulshoff L van den Broek D Bins A . Case report of a fatal serious adverse event upon administration of T cells transduced with a MART-1-specific T-cell receptor. Mol Ther. (2015) 23:1541–50. doi: 10.1038/mt.2015.60. PMID: 25896248 PMC4817886

[15] ICH . ICH International Conference on Harmonisation of Technical Requirements for Registration of Pharmaceuticals for Human Use: Preclinical Safety Evaluation of Biotechnology-Derived Pharmaceuticals. (1997). Available online at: https://database.ich.org/sites/default/files/S6_R1_Guideline_0.pdf.

[16] Bonelli M Di Giuseppe F Beken S . Impact analysis of ICH S9 on non-clinical development of anticancer drugs. Regul Toxicol Pharmacol. (2015) 73:361–6. doi: 10.1016/j.yrtph.2015.07.022. PMID: 26232707

[17] Alsalloum A Shevchenko J Sennikov S . The melanoma-associated antigen family A (MAGE-A): a promising target for cancer immunotherapy? Cancers Bsl. (2023) 15:1779. doi: 10.3390/cancers15061779. PMID: 36980665 PMC10046478

[18] Hong DS Van Tine BA Biswas S McAlpine C Johnson ML Olszanski AJ . Autologous T cell therapy for MAGE-A4+ solid cancers in HLA-A*02+ patients: a phase 1 trial. Nat Med. (2023) 29:104–14. doi: 10.1038/s41591-022-02128-z. PMID: 36624315 PMC9873554

[19] Chandora K Chandora A Saeed A Cavalcante L . Adoptive T cell therapy targeting MAGE-A4. Cancers (Basel). (2025) 17(3):413. doi: 10.3390/cancers17030413. PMID: 39941782 PMC11815873

[20] Habigt C Rottey S Spanggaard I Lopez JS Garralda E Calvo E . Mapping MAGE-A4 expression in solid cancers for targeted therapies. Front Oncol. (2025) 15:1484182. doi: 10.3389/fonc.2025.1484182. PMID: 40151801 PMC11947667

[21] Schaefer W Regula JT Bähner M Schanzer J Croasdale R Dürr H . Immunoglobulin domain crossover as a generic approach for the production of bispecific IgG antibodies. Proc Natl Acad Sci USA. (2011) 108:11187–92. doi: 10.1073/pnas.1019002108. PMID: 21690412 PMC3131342

[22] Ridgway JB Presta LG Carter P . Knobs-into-holes” engineering of antibody CH3 domains for heavy chain heterodimerization. Protein Eng. (1996) 9:617–22. doi: 10.1093/protein/9.7.617, PMID: 8844834

[23] Schlothauer T Herter S Koller CF Grau-Richards S Steinhart V Spick C . Novel human IgG1 and IgG4 Fc-engineered antibodies with completely abolished immune effector functions. Protein Eng Des Sel. (2016) 29:457–66. doi: 10.1093/protein/gzw040. PMID: 27578889

[24] Burns GF . Monoclonal antibodies human T cell subsets. J Immunol. (1982) 128:1591–6. doi: 10.1128/iai.71.12.6775-6783.2003. PMID: 14638763 PMC308885

[25] Zhu Z . Identification heavy chain residues humanized anti-CD3 antibody variant determine binding affinity. J Immunol. (1995) 154:1949–57. 7636241

[26] Betts A Keunecke A van Steeg TJ van der Graaf PH Avery LB Jones H . Linear pharmacokinetic parameters for monoclonal antibodies are similar within a species and across different pharmacological targets: a comparison between human, cynomolgus monkey and hFcRn Tg32 transgenic mouse using a population-modeling approach. MAbs. (2018) 10:751–64. doi: 10.1080/19420862.2018.1462429. PMID: 29634430 PMC6150614

[27] Ryman JT Meibohm B . Pharmacokinetics of monoclonal antibodies. CPT Pmx Syst Pharmacol. (2017) 6:576–88. doi: 10.1002/psp4.12224. PMID: 28653357 PMC5613179

[28] Van De Vyver A Eigenmann M Ovacik M Pohl C Herter S Weinzierl T . A novel approach for quantifying the pharmacological activity of T-cell engagers utilizing *in vitro* time course experiments and streamlined data analysis. AAPS J. (2021) 24:7. doi: 10.1208/s12248-021-00637-2. PMID: 34862519 PMC8817205

[29] Saber H Del Valle P Ricks TK Leighton JK . An FDA oncology analysis of CD3 bispecific constructs and first-in-human dose selection. Regul Toxicol Pharmacol. (2017) 90:144–52. doi: 10.1016/j.yrtph.2017.09.001. PMID: 28887049

[30] Aubry F Satie AP Rioux-Leclercq N Rajpert-De Meyts E Spagnoli GC Chomez P . MAGE-A4, a germ cell specific marker, is expressed differentially in testicular tumors. Cancer. (2001) 92:2778–85. doi: 10.1002/1097-0142(20011201)92:11<2778::aid-cncr10125>3.0.co;2-s 11753951

[31] Melo DH Mamede RCM Neder L Saggioro FP Figueiredo DLA da Silva WJ . Expression of MAGE-A4 and MAGE-C1 tumor-associated antigen in benign and Malignant thyroid diseases. Head Neck. (2011) 33:1426–32. doi: 10.1002/hed.21616. PMID: 21246638

[32] Freeth J Soden J . New advances in cell microarray technology to expand applications in target deconvolution and off-target screening. SLAS Discov. (2020) 25:223–30. doi: 10.1177/2472555219897567. PMID: 31885307

[33] Marrer-Berger E Nicastri A Augustin A Kramar V Liao H Hanisch LJ . The physiological interactome of TCR-like antibody therapeutics in human tissues. Nat Commun. (2024) 15:3271. doi: 10.1038/s41467-024-47062-5. PMID: 38627373 PMC11021511

[34] Smith EL Harrington K Staehr M Masakayan R Jones J Long TJ . GPRC5D is a target for the immunotherapy of multiple myeloma with rationally designed CAR T cells. Sci Transl Med. (2019) 11:eaau7746. doi: 10.1126/scitranslmed.aau7746. PMID: 30918115 PMC7508042

[35] Magré L Verstegen MMA Buschow S van der Laan LJW Peppelenbosch M Desai J . Emerging organoid-immune co-culture models for cancer research: from oncoimmunology to personalized immunotherapies. J Immunother Cancer. (2023) 11:e006290. doi: 10.1136/jitc-2022-006290, PMID: 37220953 PMC10231025

[36] Gu Z Wu Q Shang B Zhang K Zhang W . Organoid co-culture models of the tumor microenvironment promote precision medicine. Cancer Innov. (2024) 3:e101. doi: 10.1002/cai2.101. PMID: 38948532 PMC11212345

[37] Sabir HJ Nehlin JO Qanie D Harkness L Prokhorova TA Blagoev B . Separate developmental programs for HLA-A and -B cell surface expression during differentiation from embryonic stem cells to lymphocytes, adipocytes and osteoblasts. PloS One. (2013) 8:e54366. doi: 10.1371/journal.pone.0054366. PMID: 23349864 PMC3548781

[38] Isa A Nehlin JO Sabir HJ Andersen TE Gaster M Kassem M . Impaired cell surface expression of HLA-B antigens on mesenchymal stem cells and muscle cell progenitors. PloS One. (2010) 5:e10900. doi: 10.1371/journal.pone.0010900. PMID: 20531935 PMC2878340

[39] Dijkstra KK Cattaneo CM Weeber F Chalabi M van de Haar J Fanchi LF . Generation of tumor-reactive T cells by co-culture of peripheral blood lymphocytes and tumor organoids. Cell. (2018) 174:1586–1598.e12. doi: 10.1016/j.cell.2018.07.009. PMID: 30100188 PMC6558289

[40] Sachs N Papaspyropoulos A Zomer-van Ommen DD Heo I Böttinger L Klay D . Long-term expanding human airway organoids for disease modeling. EMBO J. (2019) 38:e100300. doi: 10.1101/318444 30643021 PMC6376275

[41] Harter MF Recaldin T Gjorevski N . Organoids as models of immune-organ interaction. Cell Rep. (2025) 44:116214. doi: 10.1016/j.celrep.2025.116214. PMID: 40906560

[42] Maulana TI Teufel C Cipriano M Roosz J Lazarevski L van den Hil FE . Breast cancer-on-chip for patient-specific efficacy and safety testing of CAR-T cells. Cell Stem Cell. (2024) 31:989–1002.e9. doi: 10.1016/j.stem.2024.04.018. PMID: 38754430

[43] Maulana TI Wevers NR Kristoforus T Chandler M Lanz HL Joore J . Opportunities for microphysiological systems in toxicity testing of new drug modalities. Annu Rev Pharmacol Toxicol. (2025) 65:47–69. doi: 10.1146/annurev-pharmtox-061724-080621. PMID: 39227343

[44] Esfahani K Elkrief A Calabrese C Lapointe R Hudson M Routy B . Moving towards personalized treatments of immune-related adverse events. Nat Rev Clin Oncol. (2020) 17:504–15. doi: 10.1038/s41571-020-0352-8. PMID: 32246128

[45] Yin Q Wu L Han L Zheng X Tong R Li L . Immune-related adverse events of immune checkpoint inhibitors: a review. Front Immunol. (2023) 14:1167975. doi: 10.3389/fimmu.2023.1167975. PMID: 37304306 PMC10247998

[46] Mehra T Dongre K Boesing M Frei P Suenderhauf C Zippelius A . Pre-treatment comorbidities, C-reactive protein and eosinophil count, and immune-related adverse events as predictors of survival with checkpoint inhibition for multiple tumour entities. Cancer Med. (2023) 12:12253–62. doi: 10.1002/cam4.5919. PMID: 37084178 PMC10278511

[47] Garmpis D Hidalgo-Gadea G Mauch C Tietze JK Franklin C . Combining immune-related adverse events and inflammatory profiles enhances prognostic accuracy in metastatic melanoma under PD-1-based therapy. Front Immunol. (2025) 16:1683533. doi: 10.3389/fimmu.2025.1683533. PMID: 41103410 PMC12521244

[48] Clark RA Chong BF Mirchandani N Yamanaka K-I Murphy GF Dowgiert RK . A novel method for the isolation of skin resident T cells from normal and diseased human skin. J Invest Dermatol. (2006) 126:1059–70. doi: 10.1038/sj.jid.5700199. PMID: 16484986

[49] Kumar BV Ma W Miron M Granot T Guyer RS Carpenter DJ . Human tissue-resident memory T cells are defined by core transcriptional and functional signatures in lymphoid and mucosal sites. Cell Rep. (2017) 20:2921–34. doi: 10.2139/ssrn.3155546. PMID: 28930685 PMC5646692

[50] Gebhardt T Wakim LM Eidsmo L Reading PC Heath WR Carbone FR . Memory T cells in nonlymphoid tissue that provide enhanced local immunity during infection with herpes simplex virus. Nat Immunol. (2009) 10:524–30. doi: 10.1038/ni.1718. PMID: 19305395

[51] Cheuk S Schlums H Gallais Sérézal I Martini E Chiang SC Marquardt N . CD49a expression defines tissue-resident CD8+ T cells poised for cytotoxic function in human skin. Immunity. (2017) 46:287–300. doi: 10.21417/b76k56. PMID: 28214226 PMC5337619

[52] Rashighi M Harris JE . Interfering with the IFN-γ/CXCL10 pathway to develop new targeted treatments for vitiligo. Ann Transl Med. (2015) 3:343. doi: 10.1016/b978-0-7020-6912-3.00253-6. PMID: 26734651 PMC4690998

[53] Ribot JC Lopes N Silva-Santos B . γδ T cells in tissue physiology and surveillance. Nat Rev Immunol. (2021) 21:221–32. doi: 10.1038/s41577-020-00452-4. PMID: 33057185

[54] Hayday A Dechanet-Merville J Rossjohn J Silva-Santos B . Cancer immunotherapy by γδ T cells. Science. (2024) 386:eabq7248. doi: 10.1126/science.abq7248. PMID: 39361750 PMC7616870

[55] Recaldin T Steinacher L Gjeta B Harter MF Adam L Kromer K . Human organoids with an autologous tissue-resident immune compartment. Nature. (2024) 633:165–73. doi: 10.1038/s41586-024-07791-5. PMID: 39143209 PMC11374719

[56] Steinacher L Gjeta B Mendes MP Cremasco F Cubela I Bellavista M . Human lung alveolar model with an autologous innate and adaptive immune compartment. (2025), 2025.02.27.640440. doi: 10.1101/2025.02.27.640440, PMID: 41887800

[57] Adaptimmune announces U.s. fda acceptance of biologics license application for Afami-cel for the treatment of advanced synovial sarcoma with priority review. Adaptimmune Therapeutics PLC. (2024). Available online at: https://www.adaptimmune.com/investors-and-media/news-center/press-releases/detail/260/adaptimmune-announces-u-s-fda-acceptance-of-biologics (Accessed October 30, 2025).

[58] D’Angelo SP Araujo DM Abdul Razak AR Agulnik M Attia S Blay J-Y . Afamitresgene autoleucel for advanced synovial sarcoma and myxoid round cell liposarcoma (SPEARHEAD-1): an international, open-label, phase 2 trial. Lancet. (2024) 403:1460–71. doi: 10.1891/9780826148537.0025. PMID: 38554725 PMC11419333

[59] Lin Y-J Lin Z . *In vitro*-in silico-based probabilistic risk assessment of combined exposure to bisphenol A and its analogues by integrating ToxCast high-throughput *in vitro* assays with *in vitro* to *in vivo* extrapolation (IVIVE) via physiologically based pharmacokinetic (PBPK) modeling. J Haz Mater. (2020) 399:122856. doi: 10.1016/j.jhazmat.2020.122856. PMID: 32937695

[60] Bryniarski MA Tuhin MTH Acker TM Wakefield DL Sethaputra PG Cook KD . Cellular neonatal Fc receptor recycling efficiencies can differentiate target-independent clearance mechanisms of monoclonal antibodies. J Pharm Sci. (2024) 113:2879–94. doi: 10.1016/j.xphs.2024.06.013. PMID: 38906252

[61] Bryniarski MA Tuhin MTH Shomin CD Nasrollahi F Ko EC Soto M . Utility of cellular measurements of non-specific endocytosis to assess the target-independent clearance of monoclonal antibodies. J Pharm Sci. (2024) 113:3100–11. doi: 10.1016/j.xphs.2024.07.009. PMID: 39009346

[62] Grevys A Nilsen J Sand KMK Daba MB Øynebråten I Bern M . A human endothelial cell-based recycling assay for screening of FcRn targeted molecules. Nat Commun. (2018) 9:621. doi: 10.1038/s41467-018-03061-x. PMID: 29434196 PMC5809500

[63] Kraft TE Richter WF Emrich T Knaupp A Schuster M Wolfert A . Heparin chromatography as an *in vitro* predictor for antibody clearance rate through pinocytosis. MAbs. (2020) 12. doi: 10.1080/19420862.2019.1683432. PMID: 31769731 PMC6927760

[64] Zhou Y Dai Q Xu Y Wu S Cheng M Zhao B . PharmaFormer predicts clinical drug responses through transfer learning guided by patient derived organoid. NPJ Precis Oncol. (2025) 9:282. doi: 10.1038/s41698-025-01082-6. PMID: 40804292 PMC12350944

[65] Edwards M Blanquie O Ehmann F . Insights into new approach methodology innovation: an EMA perspective. Nat Rev Drug Discov. (2025) 24:325–6. doi: 10.1038/d41573-025-00052-8. PMID: 40128616

[66] FDA pushes to replace animal testing. Nat Biotechnol. (2025) 43:655. doi: 10.1038/s41587-025-02690-0. PMID: 40380010

