# Supplementary figures and images for: Translational strategy to support the first-in-human study of a TCR-like T cell bispecific with an in vitro-based safety approach
Source: Front Immunol. 2026 Apr 17;17:1736584. doi: 10.3389/fimmu.2026.1736584 (PMC13133561; doi:10.3389/fimmu.2026.1736584)

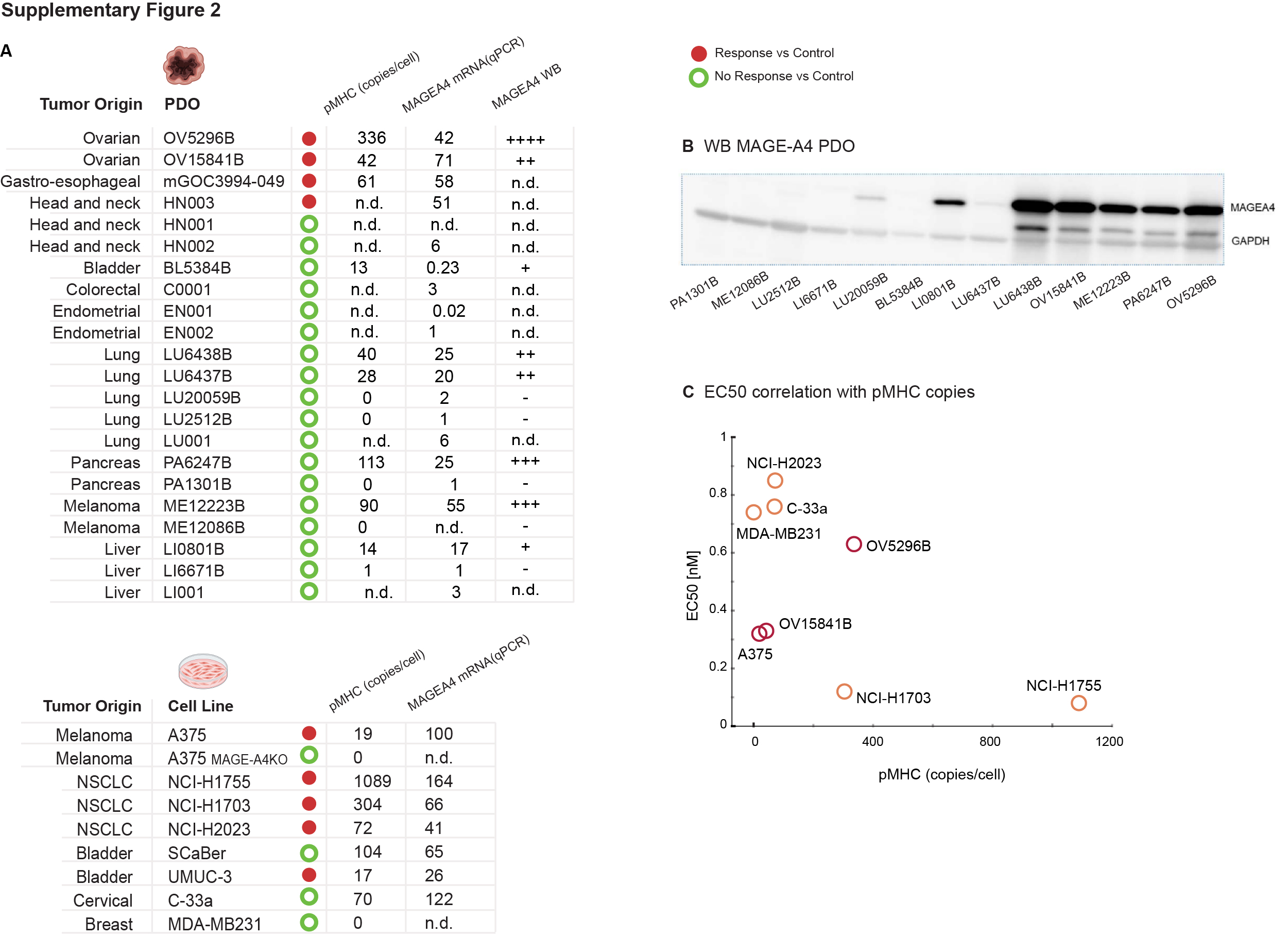

Supplement: Supplementary Figure 2 — (A) Quantification of MAGE-A4 expression in tumor models. (Left) Density of MAGE-A4/HLA-A*02:01 complexes quantified by targeted liquid chromatography-tandem mass spectrometry (LC-MS/MS) (Right) Relative MAGEA4 mRNA levels quantified by real-time qPCR, expressed as a percentage of the expression levels measured in the A375 reference cell line. Data represent the mean SD of technical replicates (n=3-4) (B) Western blot analysis of total MAGE-A4 protein levels in extracts from patient-derived organoid (PDO) lines using a rabbit monoclonal anti-MAGE-A4 antibody. Detection of GAPDH served as a loading control. (C) Correlation of target expression levels with MAGE-A4 TCB cytotoxic potency (EC50). Higher cytotoxic potency (lower EC50 values) generally correlates with higher complex densities. While efficient tumor cell lysis was observed at low complex levels in the A375 melanoma cell line (~19 copies/cell), responding PDO lines (e.g., ovarian) consistently demonstrated densities > 40 copies per cell. [file Image1.jpeg]
