# Supplementary material for: Translational strategy to support the first-in-human study of a TCR-like T cell bispecific with an in vitro-based safety approach
Source: Front Immunol. 2026 Apr 17;17:1736584. doi: 10.3389/fimmu.2026.1736584 (PMC13133561; doi:10.3389/fimmu.2026.1736584)

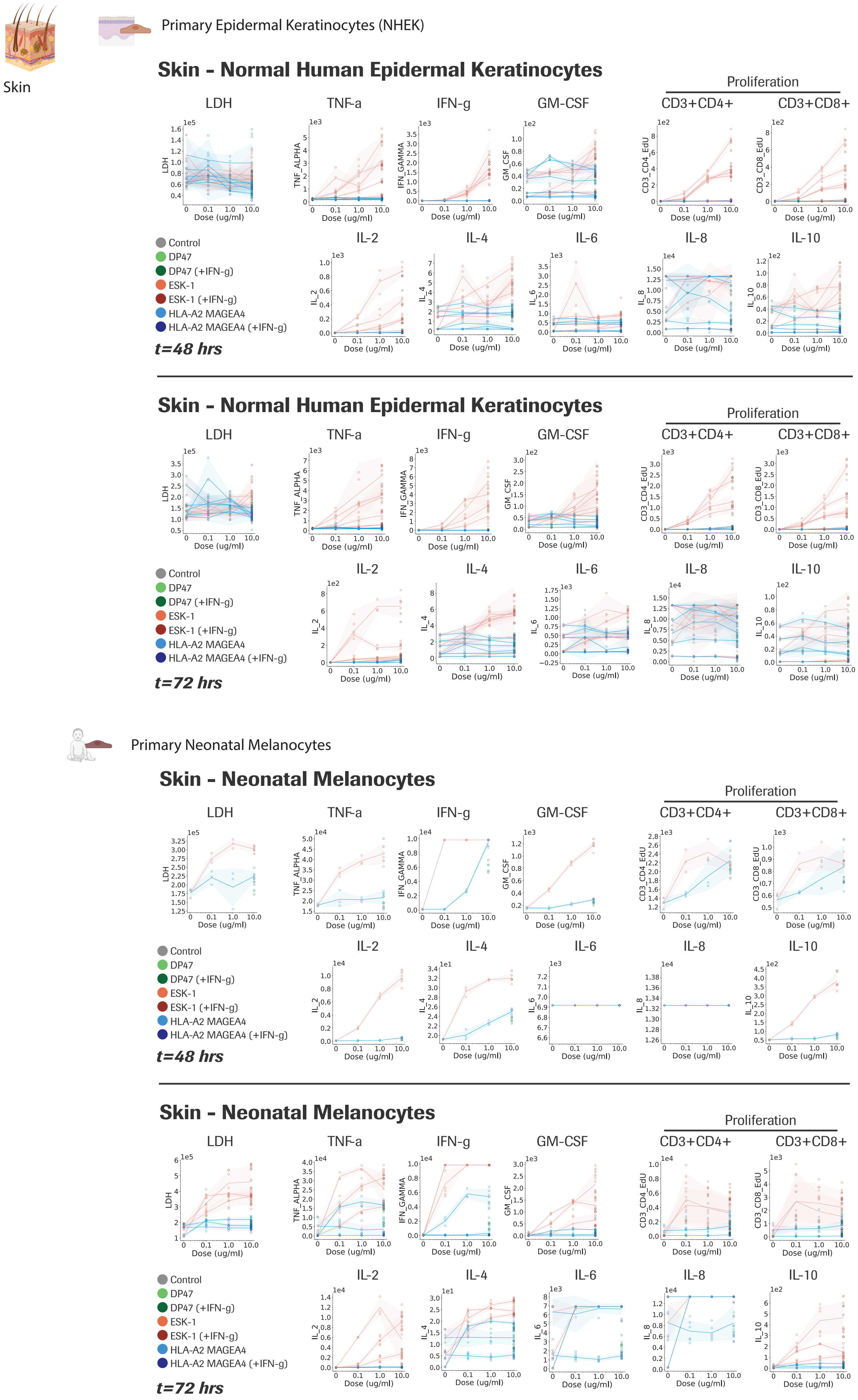

Skin
Primary Epidermal Keratinocytes (NHEK)
Skin - Normal Human Epidermal Keratinocytes
Proliferation
LDH
TNF-a
IFN-g
GM-CSF
CD3+CD4+
CD3+CD8+
IL-2
IL-4
IL-6
IL-8
IL-10
Dose (ug/ml)
Control
DP47
DP47 (+IFN-g)
ESK-1
ESK-1 (+IFN-g)
HLA-A2 MAGEA4
HLA-A2 MAGEA4 (+IFN-g)
t=48 hrs
Skin - Normal Human Epidermal Keratinocytes
Proliferation
LDH
TNF-a
IFN-g
GM-CSF
CD3+CD4+
CD3+CD8+
IL-2
IL-4
IL-6
IL-8
IL-10
Control
DP47
DP47 (+IFN-g)
ESK-1
ESK-1 (+IFN-g)
HLA-A2 MAGEA4
HLA-A2 MAGEA4 (+IFN-g)
t=72 hrs
Primary Neonatal Melanocytes
Skin - Neonatal Melanocytes
Proliferation
LDH
TNF-a
IFN-g
GM-CSF
CD3+CD4+
CD3+CD8+
IL-2
IL-4
IL-6
IL-8
IL-10
Control
DP47
DP47 (+IFN-g)
ESK-1
ESK-1 (+IFN-g)
HLA-A2 MAGEA4
HLA-A2 MAGEA4 (+IFN-g)
t=48 hrs
Skin - Neonatal Melanocytes
Proliferation
LDH
TNF-a
IFN-g
GM-CSF
CD3+CD4+
CD3+CD8+
IL-2
IL-4
IL-6
IL-8
IL-10
Control
DP47
DP47 (+IFN-g)
ESK-1
ESK-1 (+IFN-g)
HLA-A2 MAGEA4
HLA-A2 MAGEA4 (+IFN-g)
t=72 hrs

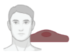

Primary Melanocytes (NHEM)

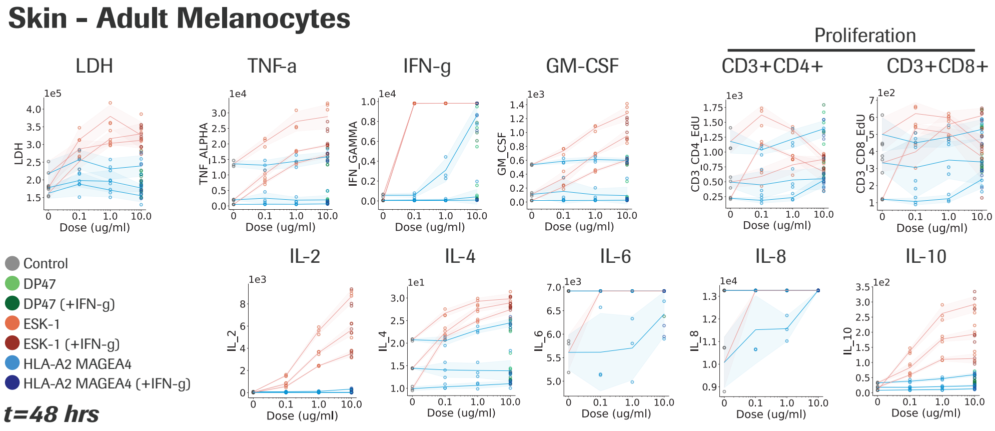


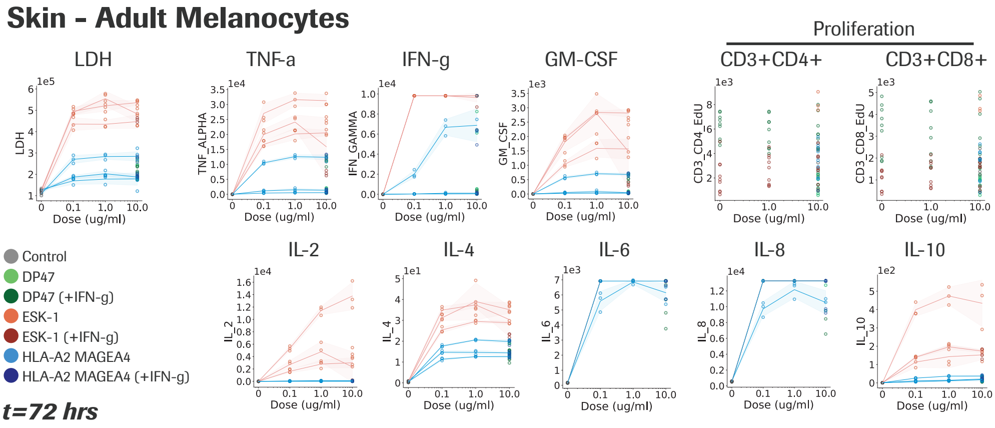

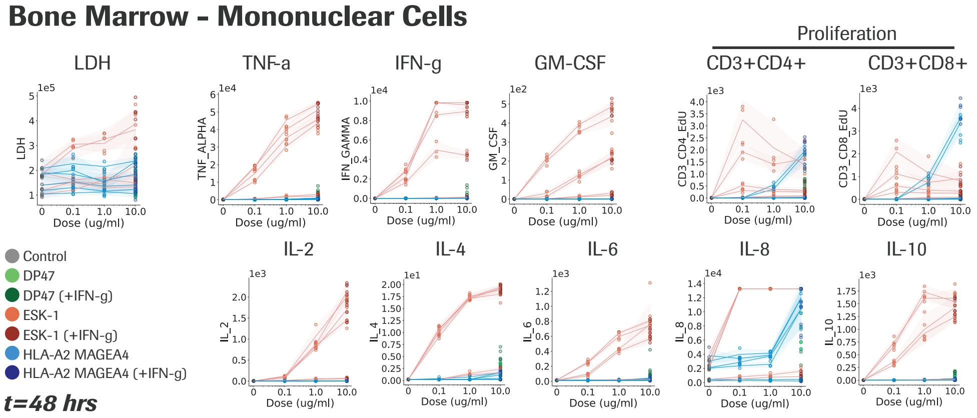
Bone Marrow - Mononuclear Cells
Proliferation
LDH
TNF-a
IFN-g
GM-CSF
CD3+CD4+
CD3+CD8+
IL-2
IL-4
IL-6
IL-8
IL-10
Dose (ug/ml)
Control
DP47
DP47 (+IFN-g)
ESK-1
ESK-1 (+IFN-g)
HLA-A2 MAGEA4
HLA-A2 MAGEA4 (+IFN-g)
t=48 hrs

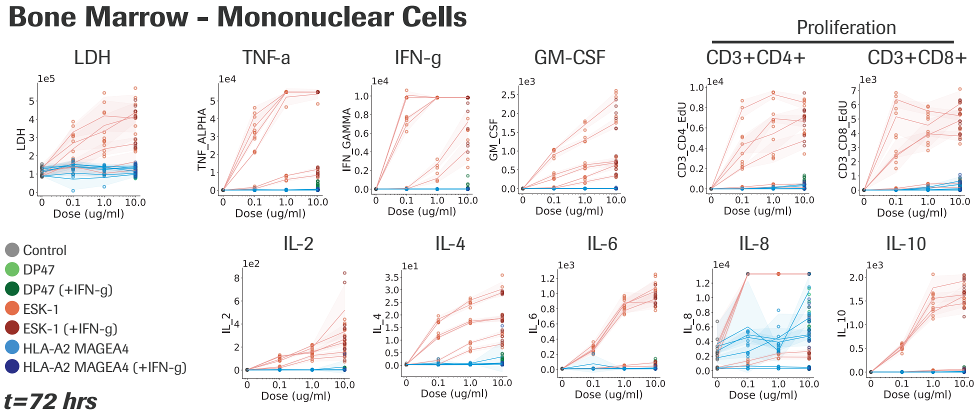
Bone Marrow - Mononuclear Cells
Proliferation
LDH
TNF-a
IFN-g
GM-CSF
CD3+CD4+
CD3+CD8+
IL-2
IL-4
IL-6
IL-8
IL-10
Dose (ug/ml)
Control
DP47
DP47 (+IFN-g)
ESK-1
ESK-1 (+IFN-g)
HLA-A2 MAGEA4
HLA-A2 MAGEA4 (+IFN-g)
t=72 hrs

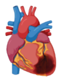

Heart

iPSC-derived beating cardiomyocytes

## iPSC-Derived Cardiomyocytes

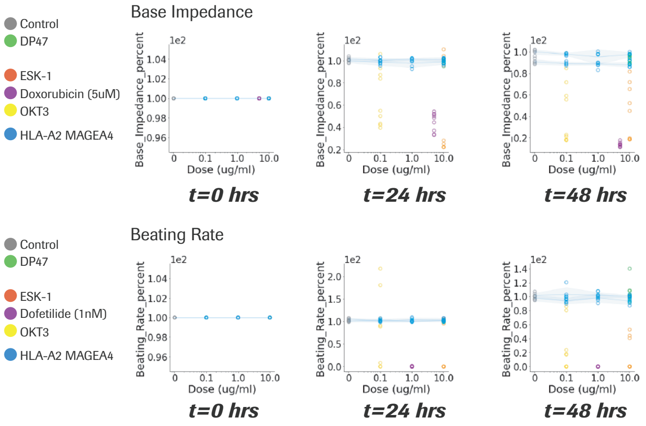


Primary Cardiac Microvascular Endothelial Cells (HMVEC-C)

## Vasculature - Microvascular Endothelial Cells

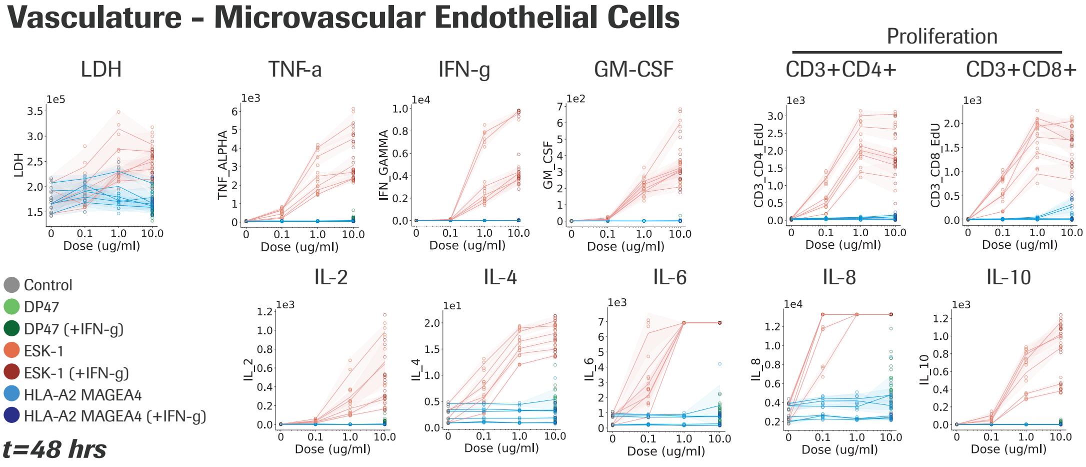


## Vasculature - Microvascular Endothelial Cells

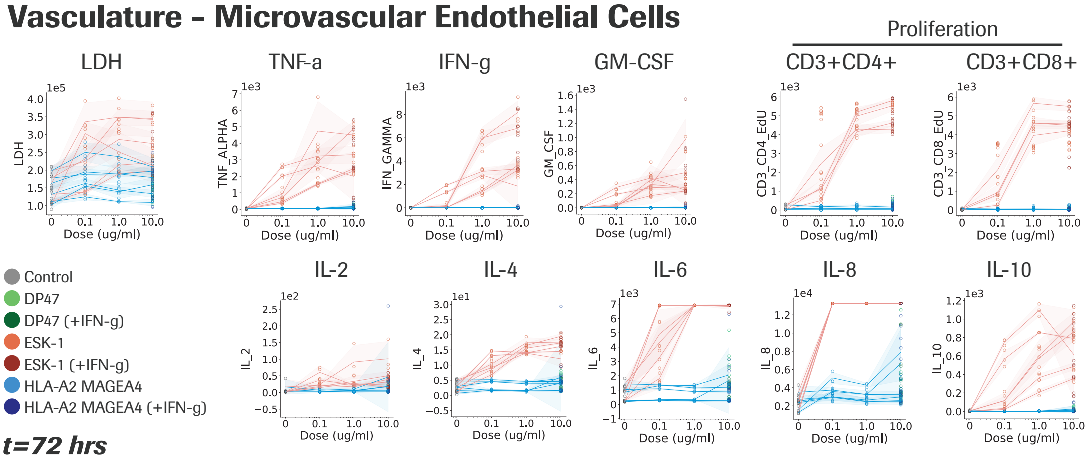

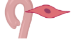

Primary Aortic Smooth Muscle Cells (AOSMC)

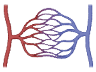

Vasculature

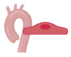

Primary Aortic Endothelial Cells (HAEC)

## Vasculature - Aortic Endothelial Cells

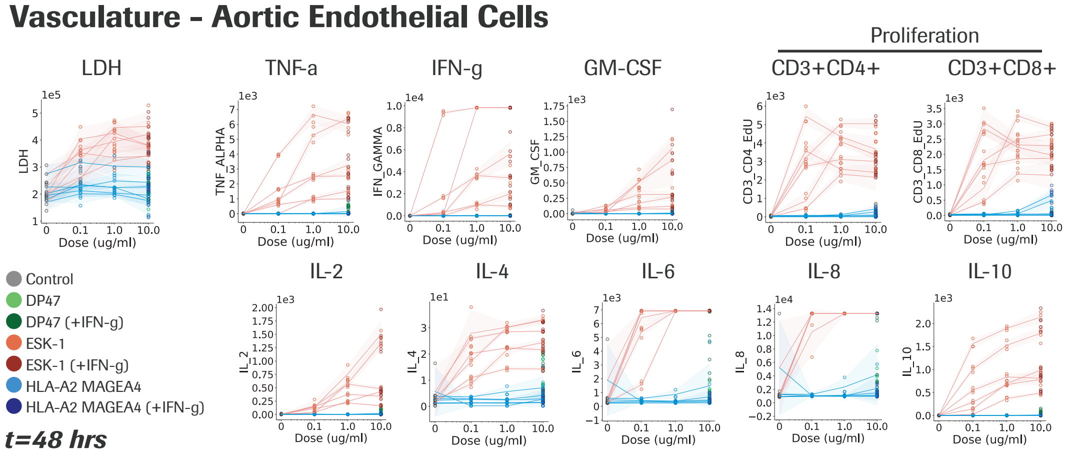


## Vasculature - Aortic Endothelial Cells

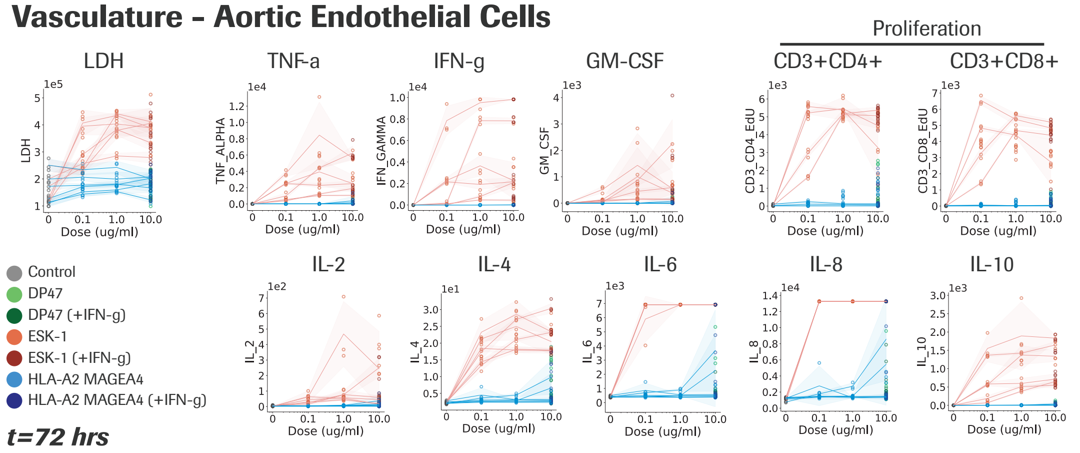


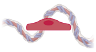

Primary Human Umbilical Vein Endothelial Cells (HUVEC)

## Human Umbilical Vein Endothelial Cells

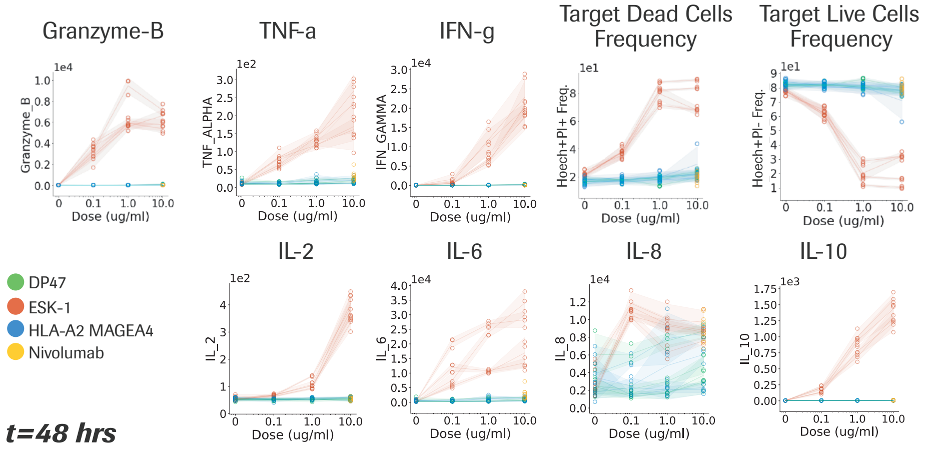

Brain

LUHMES (Lund Human Mesencephalic) neuronal cells

## Lund Human Mesencephalic Cells

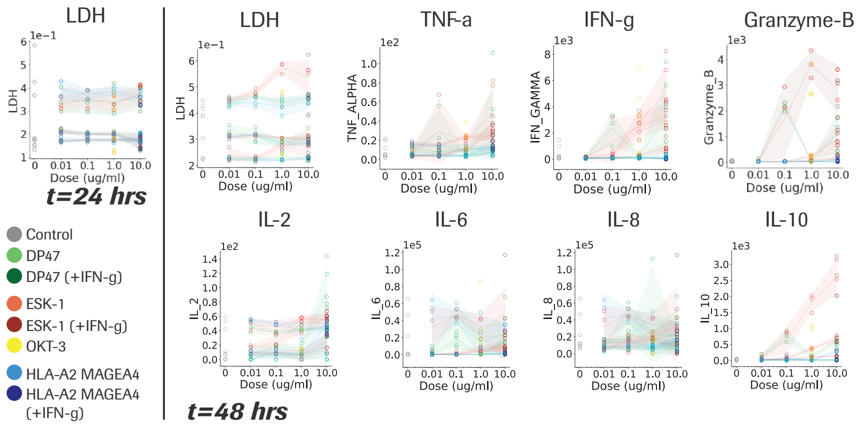


## Lund Human Mesencephalic Cells

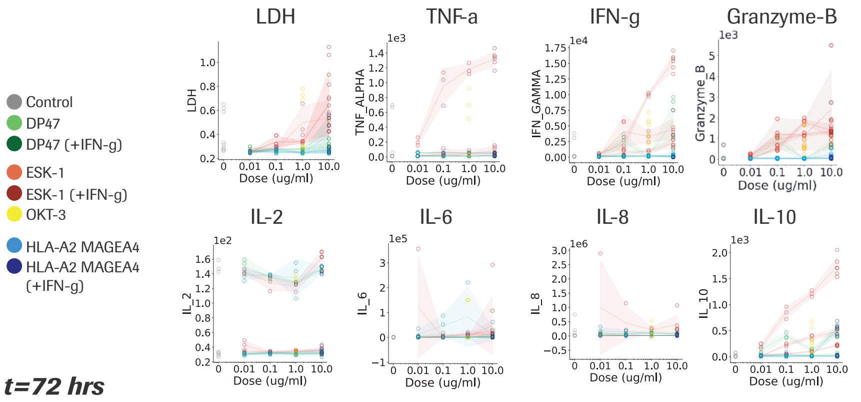


## Brain - Astrocytes

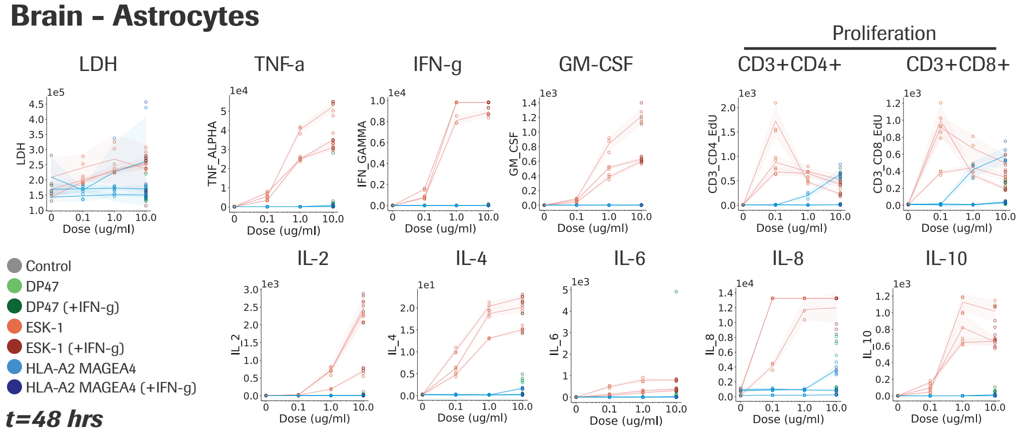


## Brain - Astrocytes

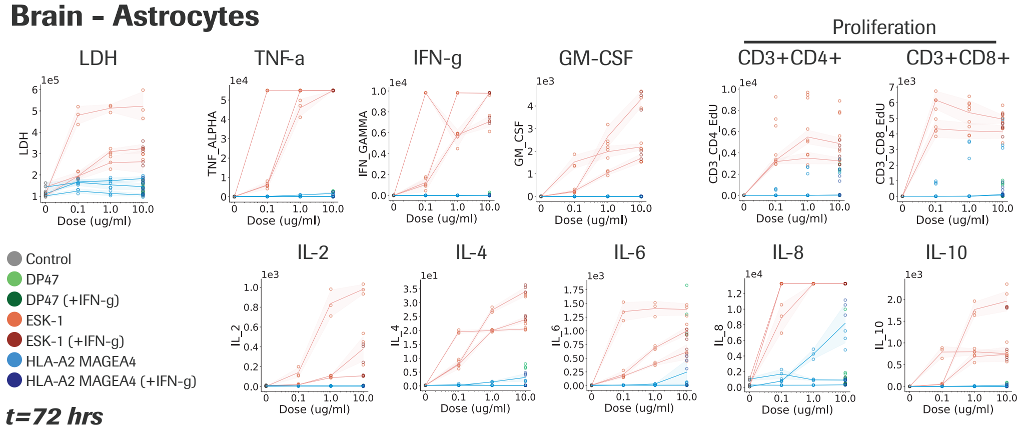

iPSC-derived astrocytes

## iPS Derived Astrocytes

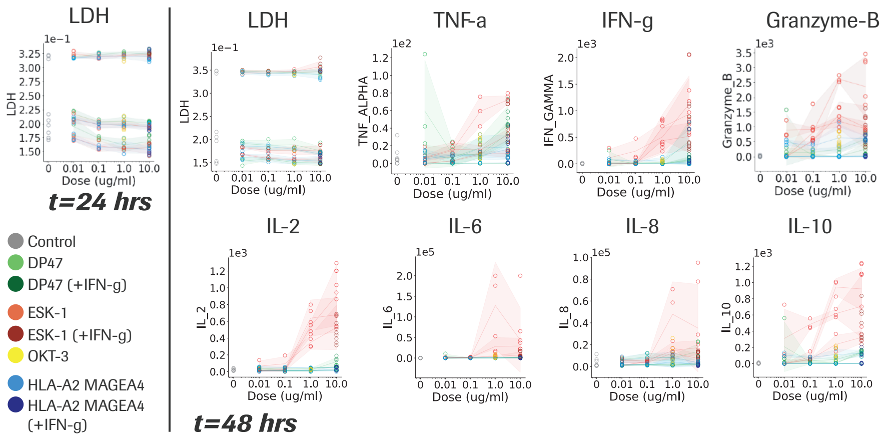


## iPS Derived Astrocytes

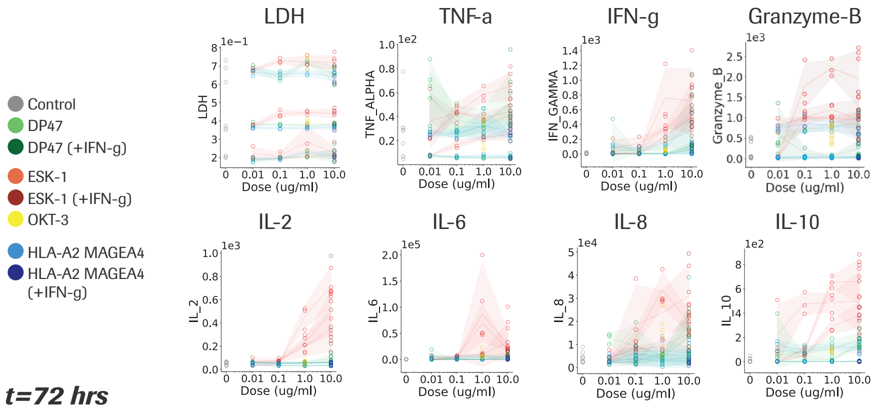


Eye

Primary Retinal Pigment Epithelial Cells (H-RPE)

## Eye – Retinal Pigment Epithelial Cells

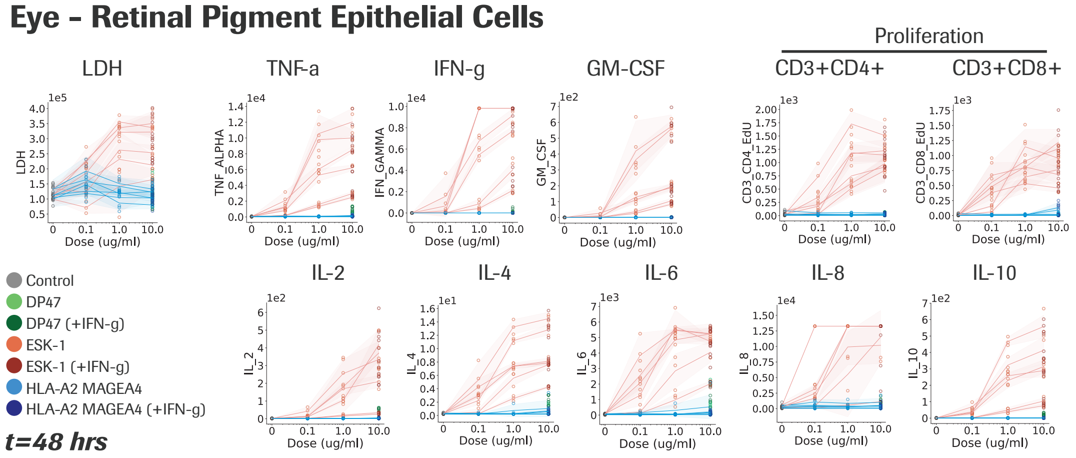


## Eye – Retinal Pigment Epithelial Cells

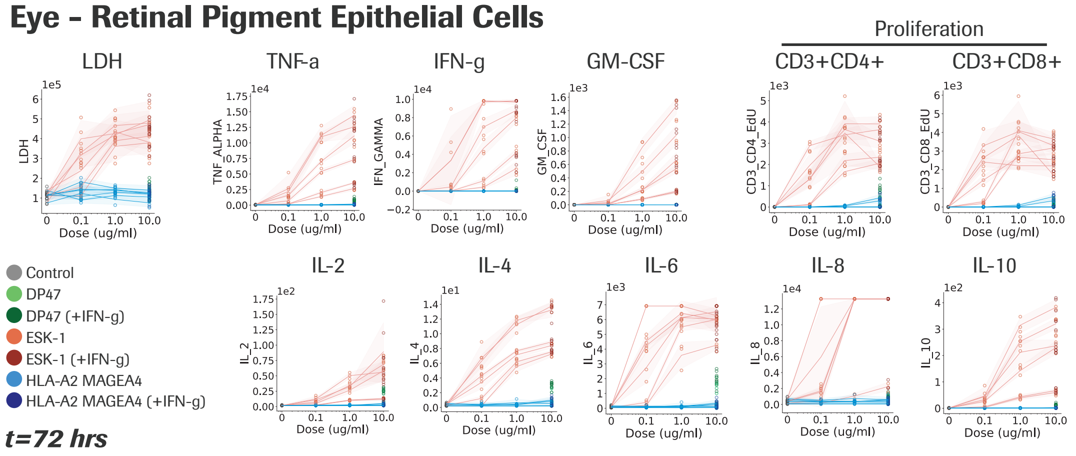

Lung Vasculature

Primary Pulmonary Artery Endothelial Cells (HPAEC)

## Vasculature - Pulmonary Artery Endothelial Cells

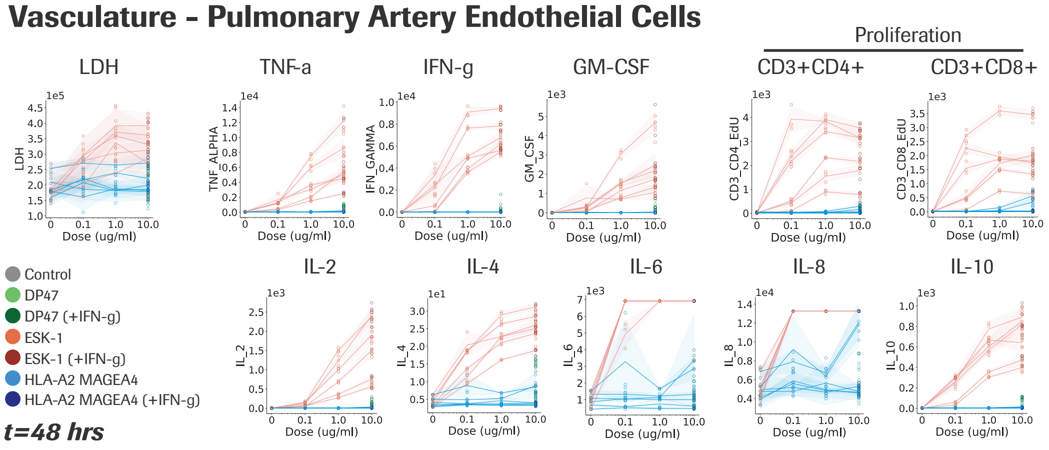


## Vasculature - Pulmonary Artery Endothelial Cells

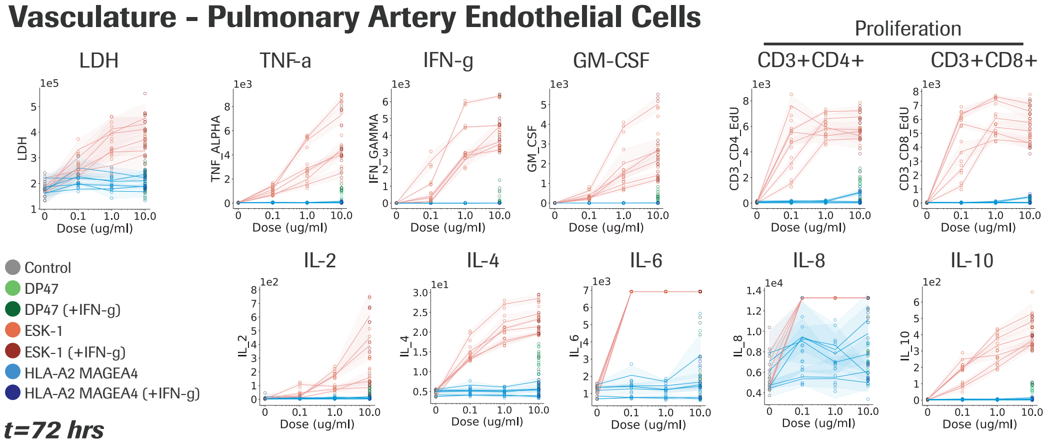

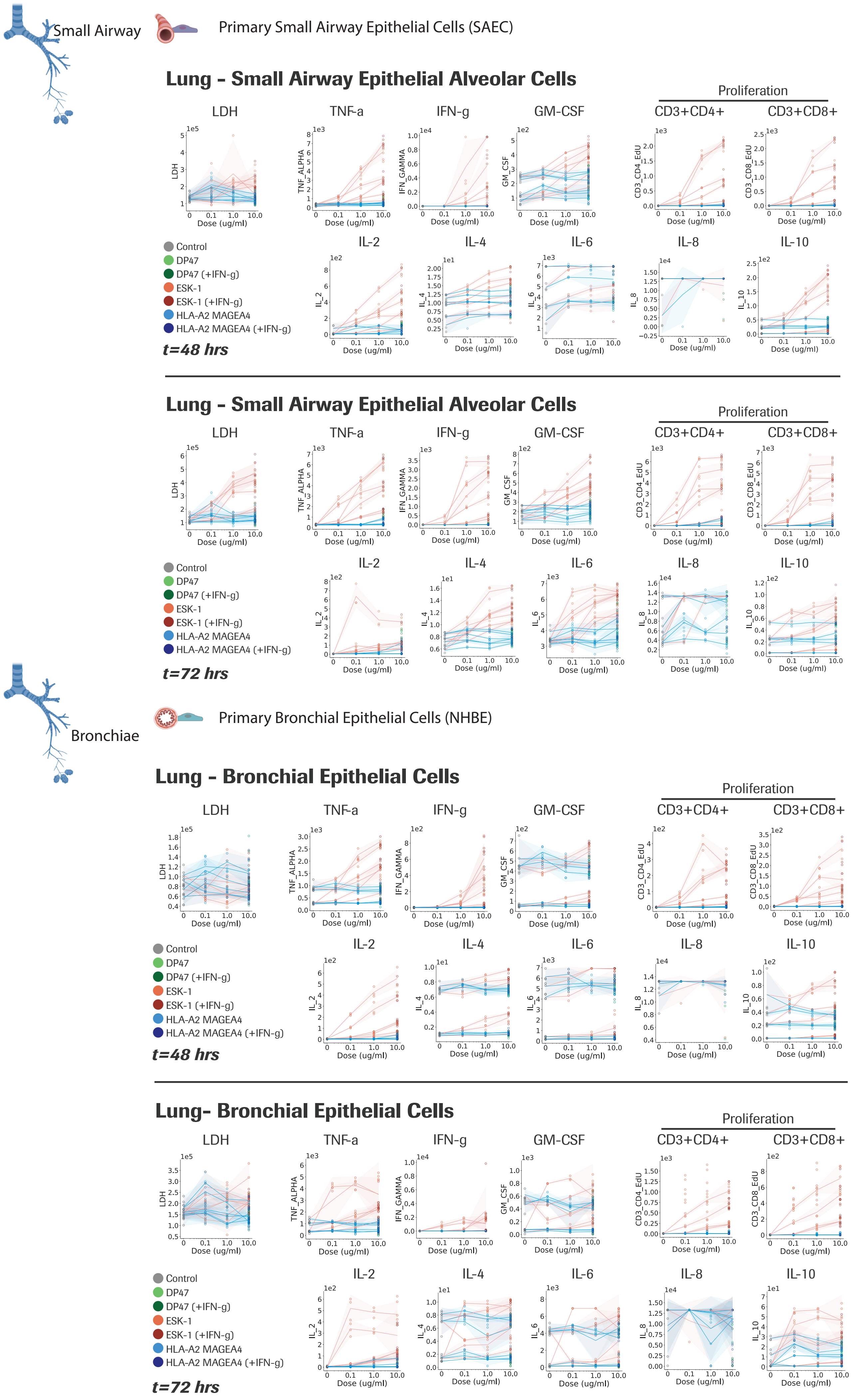

Small Airway
Primary Small Airway Epithelial Cells (SAEC)
Lung - Small Airway Epithelial Alveolar Cells
Proliferation
LDH
TNF-a
IFN-g
GM-CSF
CD3+CD4+
CD3+CD8+
IL-2
IL-4
IL-6
IL-8
IL-10
Dose (ug/ml)
Control
DP47
DP47 (+IFN-g)
ESK-1
ESK-1 (+IFN-g)
HLA-A2 MAGEA4
HLA-A2 MAGEA4 (+IFN-g)
t=48 hrs
Lung - Small Airway Epithelial Alveolar Cells
t=72 hrs
Bronchiae
Primary Bronchial Epithelial Cells (NHBE)
Lung - Bronchial Epithelial Cells
t=48 hrs
Lung- Bronchial Epithelial Cells
t=72 hrs

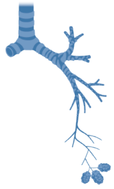

Alveoli

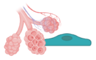

Alveolar epithelial cells Lung on a Chip (AlveoliX)

## Alveolar Epithelial Cells Type I and II - Alveolix

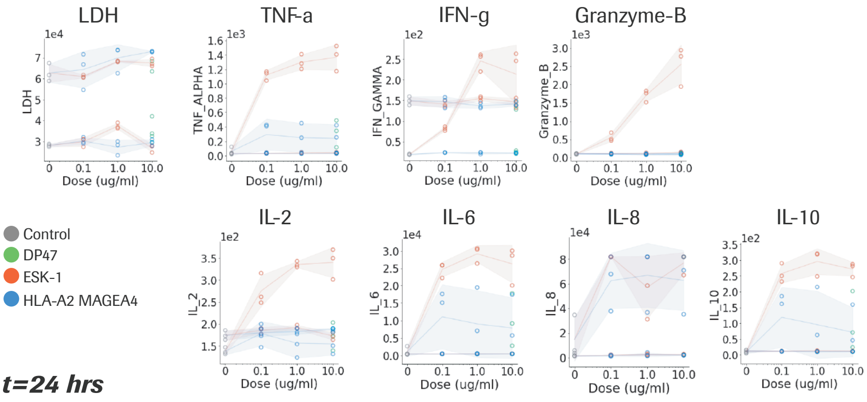


## Alveolar Epithelial Cells Type I and II - Alveolix

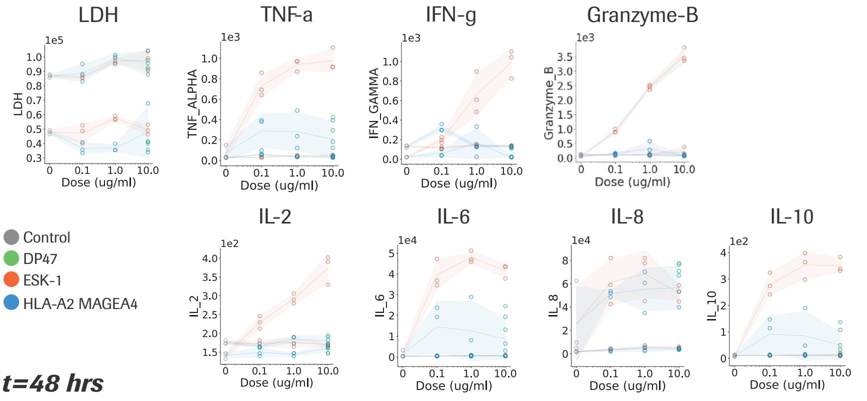


## Alveolar Epithelial Cells Type I and II - Alveolix

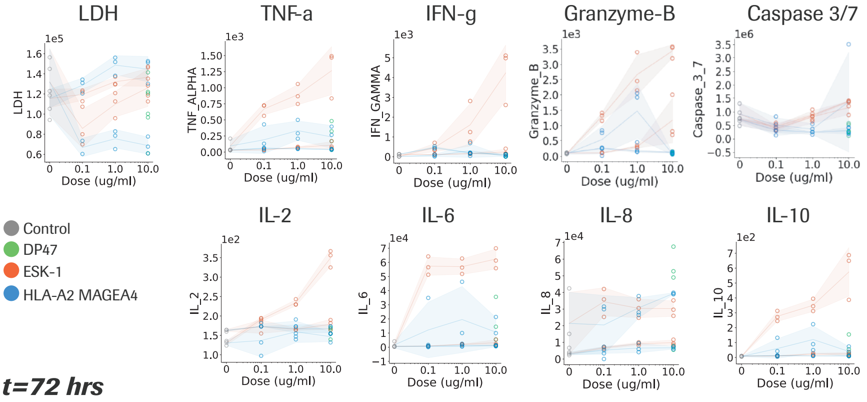

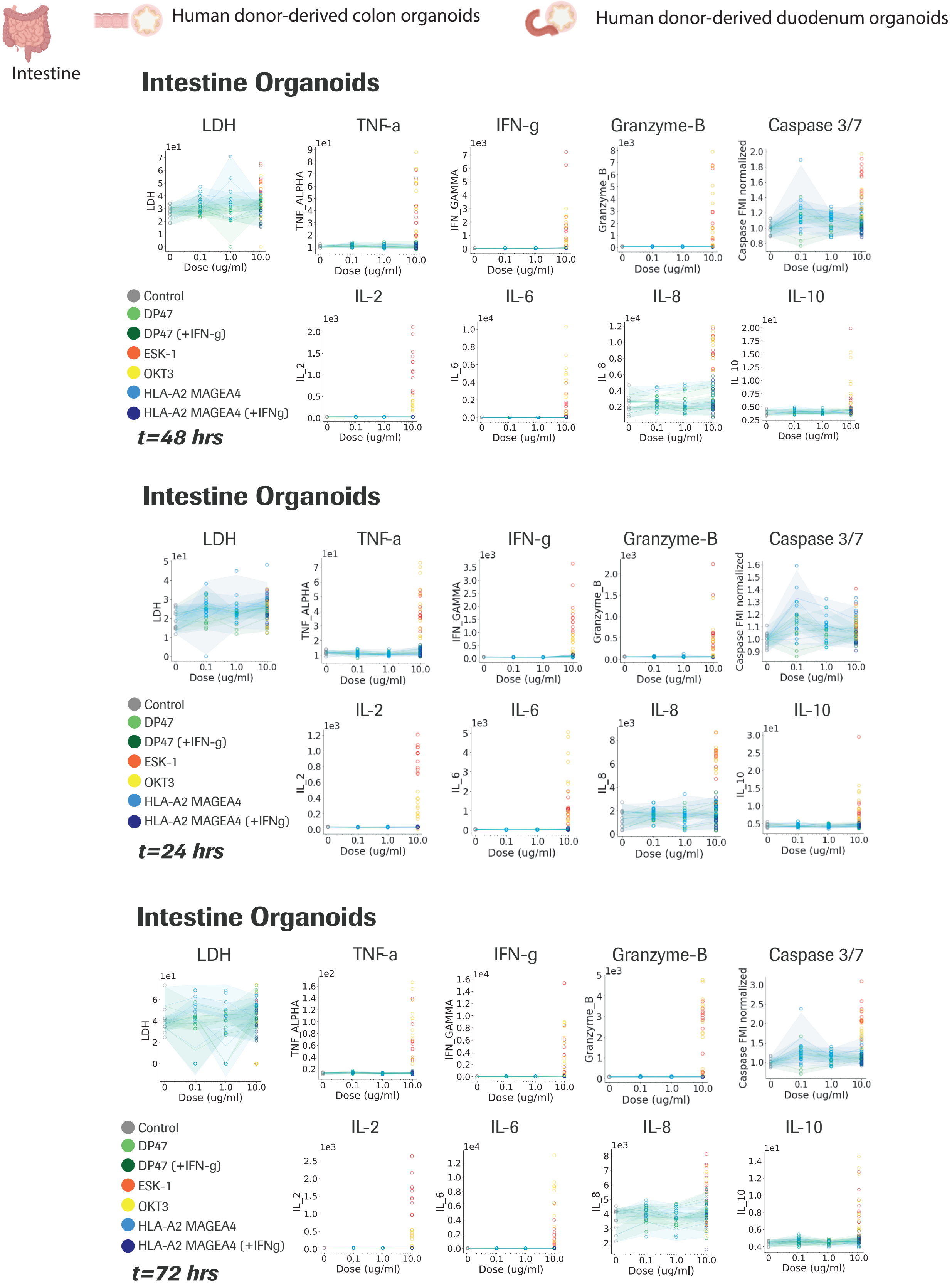
Intestine
Human donor-derived colon organoids
Human donor-derived duodenum organoids
Intestine Organoids
LDH
TNF-a
IFN-g
Granzyme-B
Caspase 3/7
IL-2
IL-6
IL-8
IL-10
Dose (ug/ml)
LDH
TNF_ALPHA
IFN_GAMMA
Granzyme_B
Caspase FMI normalized
IL_2
IL_6
IL_8
IL_10
Control
DP47
DP47 (+IFN-g)
ESK-1
OKT3
HLA-A2 MAGEA4
HLA-A2 MAGEA4 (+IFNg)
t=48 hrs
Intestine Organoids
t=24 hrs
Intestine Organoids
t=72 hrs

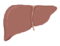
Liver
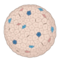
Liver spheroids - Primary human hepatocytes and non-parenchymal cells

# Liver Spheroids

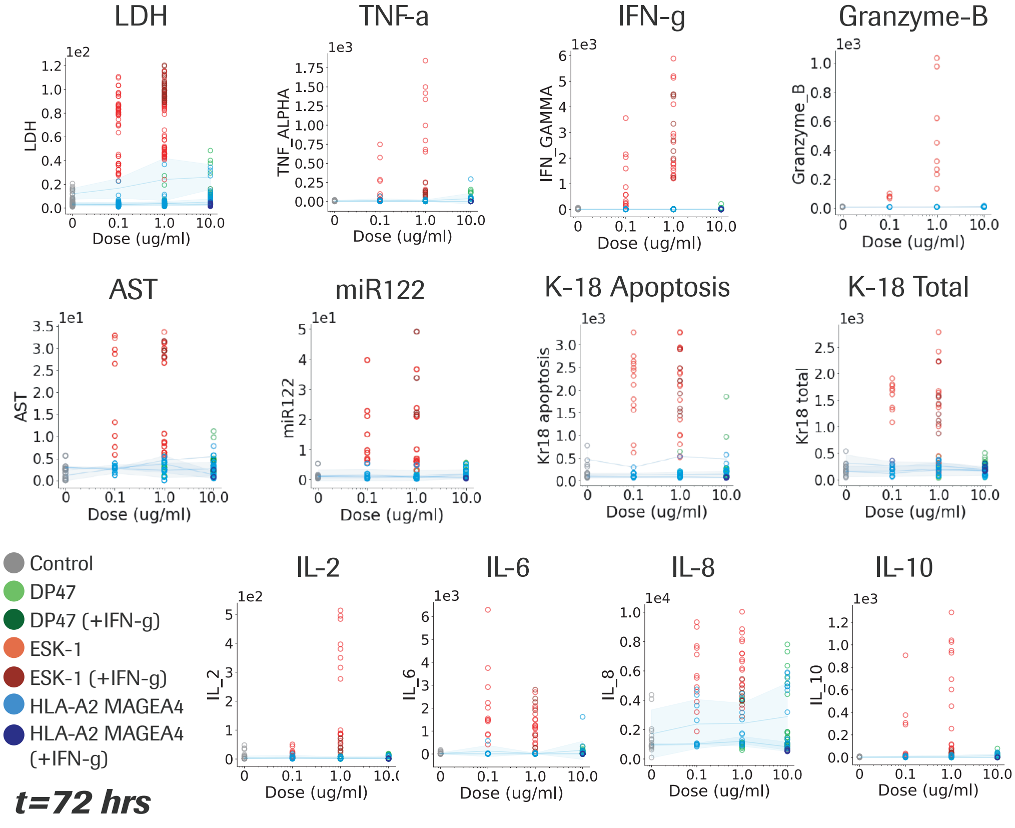


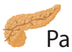
Pancreas
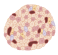
Islet Microtissues - alpha-, beta-, and delta-cells from primary human pancreatic islets of Langerhans (InSphero)

# Pancreatic Islet Spheroids

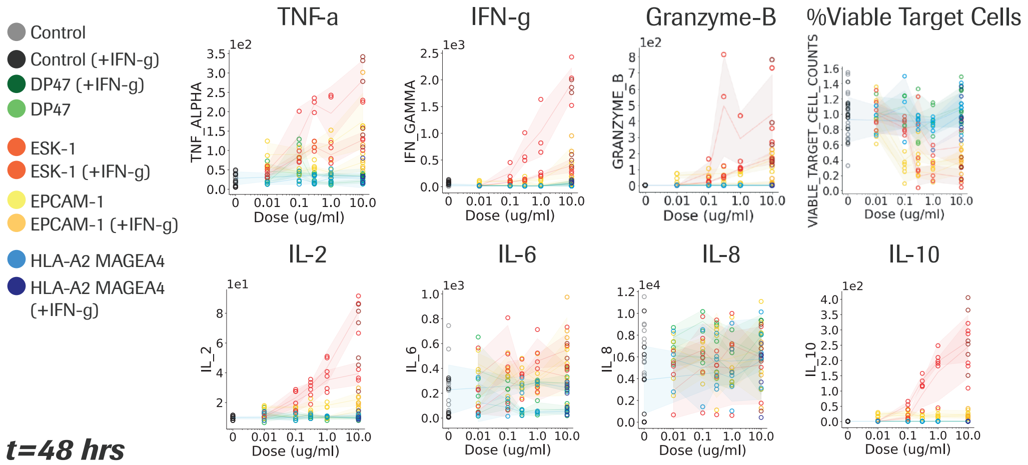

# Tumor – Lung Squamous Cell Carcinoma

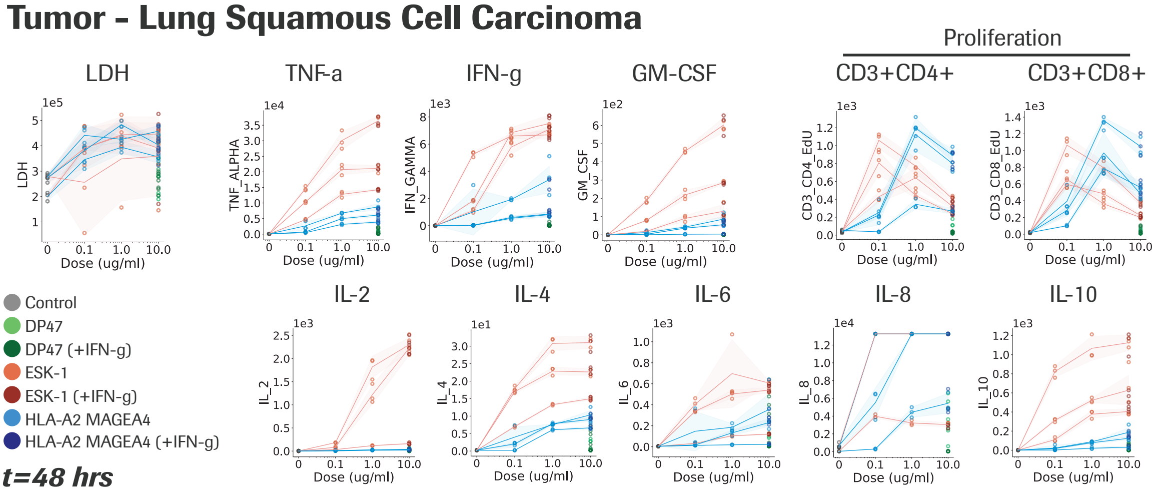


# Tumor – Lung Squamous Cell Carcinoma

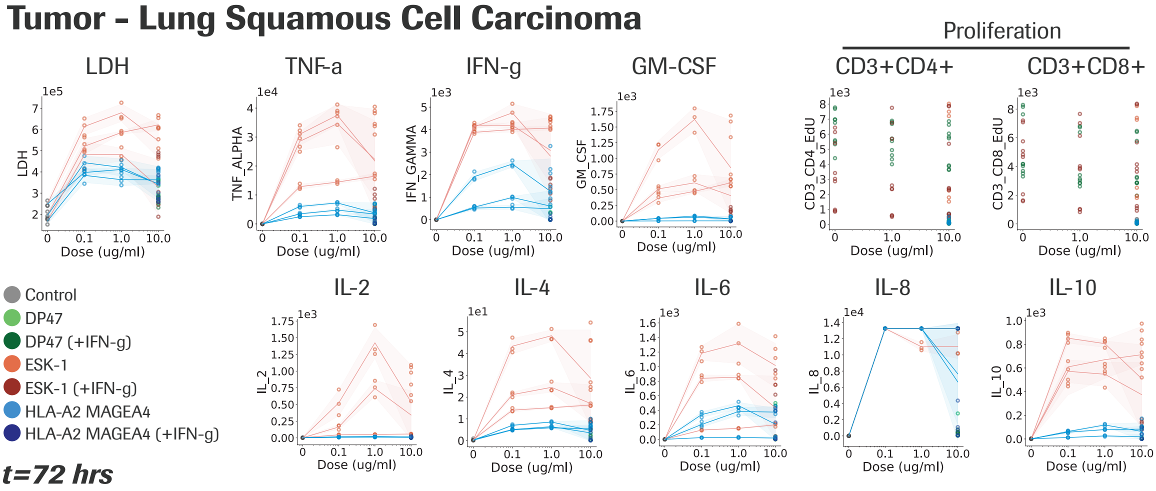

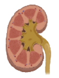


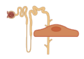

Primary Renal Epithelial Cells comprising of tubular and glomerular epithelial cells (HREpiC)

## Kidney - Renal Epithelial Cells

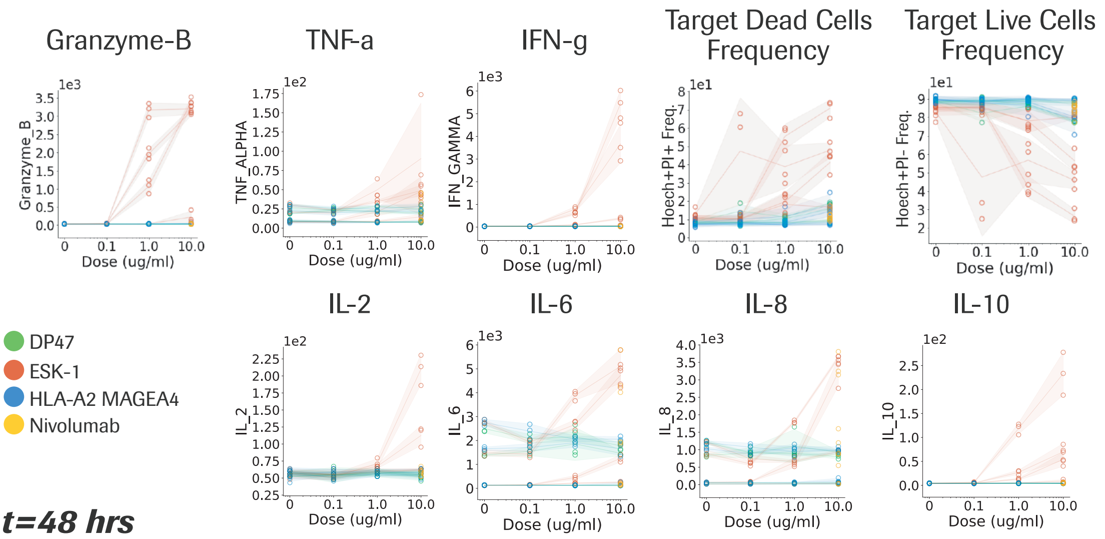


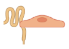

Primary Renal Proximal Tubule Epithelial Cells (RPTEC)

## Kidney - Renal Proximal Tubule Epithelial Cells

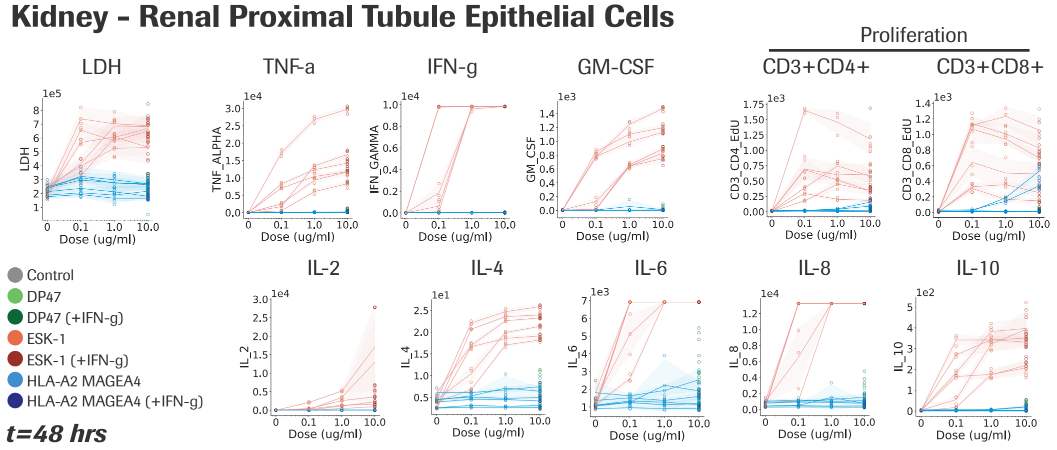


## Kidney - Renal Proximal Tubule Epithelial Cells

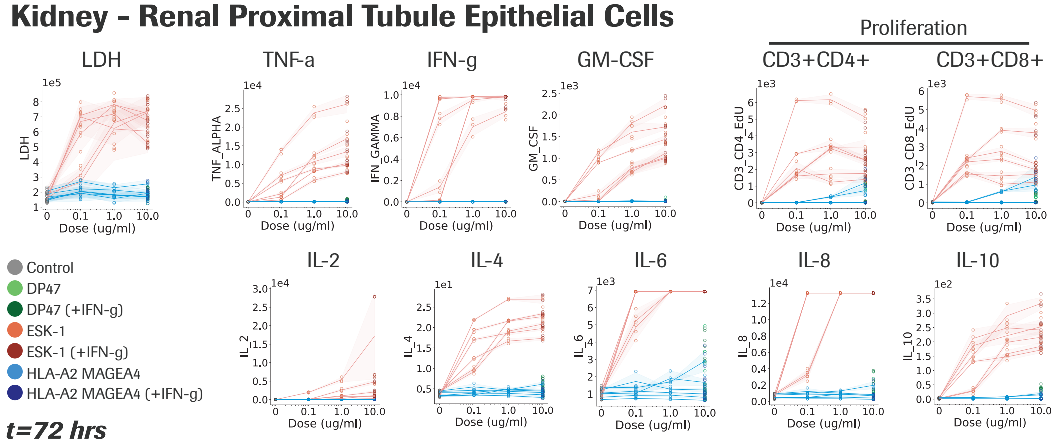

Supplement: Supplementary file 2 [file DataSheet1.pdf]
